# Evolution of the angiopoietin-like gene family in teleosts and their role in skin regeneration

**DOI:** 10.1186/s12862-016-0859-x

**Published:** 2017-01-13

**Authors:** Rita A. Costa, João C. R. Cardoso, Deborah M. Power

**Affiliations:** Comparative Endocrinology and Integrative Biology, Centre of Marine Sciences, Universidade do Algarve, Campus de Gambelas, 8005-139 Faro, Portugal

**Keywords:** Angiopoietin-like proteins, Evolution, Expression, Skin regeneration, Teleost

## Abstract

**Background:**

The skin in vertebrates is a protective barrier and damage is rapidly repaired to re-establish barrier function and maintain internal homeostasis. The angiopoietin-like (ANGPTL) proteins are a family of eight secreted glycoproteins with an important role in skin repair and angiogenesis in humans. In other vertebrates their existence and role in skin remains largely unstudied. The present study characterizes for the first time the homologues of human ANGPTLs in fish and identifies the candidates that share a conserved role in skin repair using a regenerating teleost skin model over a 4-day healing period.

**Results:**

Homologues of human *ANGPTL1-7* were identified in fish, although *ANGPTL8* was absent and a totally new family member designated *angptl9* was identified in fish and other non-mammalian vertebrates. In the teleost fishes a gene family expansion occurred but all the deduced Angptl proteins retained conserved sequence and structure motifs with the human homologues. In sea bream skin *angptl1b*, *angptl2b*, *angptl4a*, *angptl4b* and *angptl7* transcripts were successfully amplified and they were differentially expressed during skin regeneration. In the first 2 days of skin regeneration, re-establishment of the physical barrier and an increase in the number of blood vessels was observed. During the initial stages of skin regeneration *angptl1b* and *angptl2b* transcripts were significantly more abundant (*p* < 0.05) than in intact skin and *angptl7* transcripts were down-regulated (*p* < 0.05) throughout the 4-days of skin regeneration that was studied. No difference in *angptl4a* and *angptl4b* transcript abundance was detected during regeneration or between regenerating and intact skin.

**Conclusions:**

The *angptl* gene family has expanded in teleost genomes. In sea bream, changes in the expression of *angptl1b*, *angptl2b* and *angptl7* were correlated with the main phases of skin regeneration, indicating the involvement of ANGPTL family members in skin regeneration has been conserved in the vertebrates. Exploration of the fish *angptl* family in skin sheds new light on the understanding of the molecular basis of skin regeneration an issue of importance for disease control in aquaculture.

**Electronic supplementary material:**

The online version of this article (doi:10.1186/s12862-016-0859-x) contains supplementary material, which is available to authorized users.

## Background

The skin is the largest organ in the body and its role in innate immunity as a barrier between the external and internal environment makes it of major importance for the maintenance of homeostasis. This organ is well supplied with blood vessels and nerve endings that receive tactile and thermal stimuli from the environment [1]. The skin has evolved from a simple respiratory epithelium in the amphioxus [2] to a complex multicellular and multipurpose tissue in vertebrates [3, 4]. The general structure of skin in all vertebrates has been conserved and it is composed of an upper epidermal layer that is an interface with the exterior, an intermediate dermal layer and the basal hypodermal layer. Fish skin differs in several aspects from mammalian skin and the functional divergence between skin in a terrestrial and aquatic environment is presumably underpinned by significant divergence in molecular and cellular processes. While in human skin the primary physical barrier that confers protection is the stratified epidermis that is composed of dead keratinized cells, in fish the epidermis is composed of metabolically active cells with little keratinization [5–8]. Goblet cells and club cells produce mucous rich in proteases, mucins, immunoglobulins and antimicrobial peptides (AMPs) that protect the living epidermis of the fish integument. The most pronounced difference between the skin in terrestrial and aquatic vertebrates is the presence in fish skin of scales that are mineralized structures of dermal origin, that protect the underlying dermis from abrasion and damage caused by predation [9].

The importance of the skin as a protective barrier and in the maintenance of internal homeostasis means that damage has to be rapidly repaired. The process of skin repair in vertebrates is complex and involves a cascade of local and systemic responses to restore tissue integrity. In mammals the outcome of injury to skin is repair and scarring but in amphibians and fish regeneration occurs and the disrupted tissue is replaced by skin with the original tissue architecture [3]. In fish, scale removal provokes a wound and the loss of epidermal cells, scales and the superficial dermis. The removal of scales damages a key barrier of the innate immune system and consequently provokes an inflammatory response and activation of the processes associated with healing and skin and scale re-growth [5]. Fish skin heals rapidly and the wound surface is rapidly covered in mucus and re-epithelialization occurs from the wound margin [10, 11]. Skin and scale regeneration in fish involves, re-epithelialization and differentiation of scale-forming cells (day 1–2), production of the external layer matrix (days 3–5), production of the basal-plate matrix (days 6–14) and finally partial mineralization of the basal plate (days 14–28) [12]. Wound repair and skin regeneration studies are numerous in mammals [13, 14] and amphibians [15, 16], but are much less frequent in the fishes, the largest group of extant vertebrates [17] and the molecular basis of skin repair is generally restricted to single gene studies [18–21]. Recent studies have used microarrays to assess the response of fish skin to damage or ectoparasites [8, 22] and members of the angiopoietin family are among the differentially expressed genes detected.

In mammals, the ANGPTL family is composed of 8 secreted glycoproteins (ANGPTL1 to 8) that regulate a plethora of physiological and pathophysiological processes and in the skin they are involved in tissue repair and cell proliferation [23]. Members of this family are characterised by the presence of an amino-terminal coiled-coil domain (CCD), a linker region and a carboxyl-terminal fibrinogen-related domain (FReD). The exception is ANGPTL8 that is an atypical shorter family member that has lost the FReD domain and is only described in mammals [24]. ANGPTLs are structurally similar to Angiopoietins (ANGPT), an important family of vascular growth factors [25–29]. Recently it was demonstrated that some of the actions of ANGPTL are mediated by receptors that belong to the immunoglobulin-like superfamily [30]. In humans, ANGPTL4 induces keratinocyte migration during wound healing [31, 32] and epidermal differentiation post-healing [33]. In mouse, overexpression of *ANGPTL6* in skin promotes epidermal hyperplasia and enlargement of dermal lymphatic and blood vessels to favour wound healing [34, 35]. ANGPTL7 regulates extracellular matrix (ECM) formation [36] and is highly expressed in keratinocytes and is a potent anti-angiogenic factor in the cornea [37]. This protein is also described to inhibit tumour growth in a mouse xenograph model [38] and is required for the regeneration of human hematopoietic stem and progenitor cells (HSPCs) [39, 40].

The functional importance of ANGPTL in mammalian skin makes them interesting candidate molecules for skin regeneration in fish. Homologues of several mammalian ANGPTL members have been described in teleosts. In particular, orthologues of human *ANGPTL2*, human *ANGPTL7* and human *ANGPTL4* have been described respectively, in fin repair [41, 42], in the dermatome [43] and in metabolically modified skin [8] of fish. The preceding observation together with the reported role of ANGPTL in mammalian skin repair led us to hypothesize that Angptl plays a role in skin regeneration in fish. The existence of multiple members of the *ANGPTL* family in vertebrates and the deficit of knowledge about this gene family in fish made it necessary to first characterize the evolution of the *ANGPTL* gene family and gene synteny in order to identify the candidate gene family targeted in this study. To assist in designation of putative function we identified the motifs in the deduced piscine Angptl proteins that have been conserved during evolution. We then mapped the tissue distribution of gene family members using *in silico* molecular resources (EST and microarray probes) and confirmed the association of *angptl* family members with the integument by qPCR in sea bream intact skin and regenerating skin after scale removal. Taking into consideration the role of ANGPTL in tissue repair, cell proliferation and angiogenesis in mammals we correlated the expression patterns of *angptl1b*, *angptl2b*, *angptl4a*, *angptl4b* and *angptl7* with the initial phases of piscine skin regeneration to test if the function of the ANGPTL family was conserved during the evolution of the vertebrates.

## Methods

### Genome and EST database searches

Homologues of human angiopoietin-like (ANGPTL) family members were procured in 15 fish genome assemblies (Additional file 1: Table S1). Using as queries the deduced mature protein sequences of human ANGPTLs, ten teleost genomes were explored, nine of which were available from Ensembl [44], accessed in May 2015, and included: two puffer fishes (*Tetraodon nigroviridis*, *Takifugu rubripes*), stickleback (*Gasterosteus aculeatus*), Nile tilapia (*Oreochromis niloticus*), medaka (*Oryzias latipes*), platyfish (*Xiphophorus maculatus*), Atlantic cod (*Gadus morhua*), cavefish *(Astyanax mexicanus*) and zebrafish (*Danio rerio)* and the sea bass (*Dicentrarchus labrax*) assessed from the sea bass genome assembly [45]. Searches were complemented by mining additional fish genomes at Ensembl [44], accessed in May 2015, a basal ray-finned fish, the spotted gar (*Lepisosteus oculatus*), the coelacanth (*Latimeria chalumnae*) that is basal to the tetrapod lineage and a jawless fish, the marine lamprey (*Petromyzon marinus*). The genome of two cartilaginous fishes the elephant shark (*Callorhinchus milii*, http://esharkgenome.imcb.a-star.edu.sg/) and little skate (*Leucoraja erinacea*, http://skatebase.org/) were also analysed.

To assess *angptl* gene family evolution, searches were extended to genomes of terrestrial vertebrates and invertebrates (early deuterostomes, protostomes and early metazoan). This included 4 terrestrial vertebrates (the amphibian *Xenopus tropicalis*, the reptile the Anole lizard, *Anolis carolinensis*, the chicken, *Gallus gallus* and two mammalians: the marsupial opossum *Monodelphis domestica* and the placental mouse, *Mus musculus* available from Ensembl [44] and accessed in May 2015); 4 early deuterostomes (the hemichordate acorn worm, *Saccoglossus kowalevskii* [46], accessed in May 2015; the echinoderm sea urchin, *Strongylocentrotus purpuratus* [47], accessed in May 2015; the cephalochordate amphioxus, *Branchiostoma floridae* [48], accessed in May 2015; and the urochordate Ciona, *Ciona intestinalis* [44], accessed in May 2015); 11 protostomes (two annelids, *Capitela teleta* and *Helobdella robusta*; two molluscs *Crassostrea gigas* and *Lottia gigantea*; 5 arthropods the *Daphnia pulex*, *Ixodes scapularis*, *Tribolium castaneum, Drosophila melanogaster*, *Anopheles gambiae*, the nematode *Caernohabditis elegans* and the platyhelminth *Schistosoma mansoni*) and 2 early metazoans (the cnidarian, *Nematostella vectensis* and the porifera, *Amphimedon queenslandica*) were accessed from the Ensembl genomes database [44], accessed in May 2015. Searches for putative *angptl-like* transcripts for the target invertebrate species were also performed at the NCBI database [49] using the deduced protein of human ANGPTL against the species-specific nucleotide collections (nr/nt). The identity of all retrieved sequences as ANGPTL family members was confirmed by reverse blast searches against the human NCBI non-redundant protein sequence [45] database.

To aid in the identification of *angptl* candidates with a functional role in fish skin, the deduced sea bass Angptl protein sequences were used to identify *angptl* transcripts isolated from skin EST libraries using a tblastn query against the teleost EST collection [50] (taxid:32443). The EST sequence hits with e < −70 score were retained and their identity was confirmed by reverse blast against the human genome. Microarray probes modified in a sea bream skin/scale regeneration experiment [8] and a transcriptome assembly of sea bass skin (Patricia Pinto, *personal communication*) were also analysed for skin *angptl* candidates. For the skin expression studies, the *angptl* family members from the gilthead sea bream (*Sparus aurata*) were identified from the species-specific NCBI EST database subset [50] (taxid:8175) and a sea bream transcriptome assembly prepared from multiple tissues [51].

### Phylogenetic analysis

Phylogenetic analysis of fish and other metazoan Angptl family members was performed using the deduced mature protein sequences. Two hundred and twenty-six sequences including the Angptl1 to 9 and also Angpt 1, 2 and 4 sequences from 23 vertebrates including the 15 fish species and the cephalochordate representatives were used to construct the phylogenetic trees. The deduced mature protein sequences were aligned using ClustalW (v2) [52]. Gaps that resulted from the sequence alignment were removed using the AliView v 1.17.1 [53] and the edited Angpt/ Angptl protein alignment was submitted to the ProtTest 2.4 server [54] to identify the best model to study protein evolution using the Akaike Information Criterion (AIC) statistical model [54].

Phylogenetic analysis was performed using two approaches: Bayesian interference (BI) and maximum likelihood (ML). The BI tree was built in MrBayes 3.2 [55] using a JTT substitution model (Aamodel = Jones) [56] and 1.000.000 generations sampling and probability values to support tree branching. The ML method was performed with 100 bootstrap replicates to test the robustness of the phylogenetic clades in the ATGC interface (PhyML 3.0) [57]. The ML tree was built with a JTT substitution model with a fixed proportion of invariable sites value (0.008) and 4 gamma-distributed rate categories (1.272). Both BI and ML phylogenetic trees were rooted using the metazoan Angpt clade and they had similar branch topologies.

### Multiple sequence comparisons and analysis

The deduced mature proteins of the fish Angptl family were compared with human homologues to identify conserved motifs that have been maintained across vertebrates or that are characteristic of each family cluster identified by phylogenetic analysis. Alignments were performed in ClustalW (v2) [52] and manually edited using Genedoc [58] software that was also used to calculate the percent of sequence identity/similarity between fish, terrestrial vertebrates and cephalochordate homologues. Conserved domains in the fish sequences were identified using Smart [59] and UniProt [60] softwares. The mature protein sequences of the sea bream *angptl* transcripts were deduced using the ExPASy Translate Tool [61].

### Short-range gene linkage

To further confirm gene identity and to establish an evolutionary model for the metazoan *Angptl* genes, the gene environment of the chromosomes or genome fragments of vertebrate *Angptl7* was isolated to establish if a homologue genome region existed in the cephalochordate. Similarly, the gene environment of mammalian *Angptl8* was characterized to comprehend the absence of this gene in other non-mammalian vertebrates. The gene environment of the novel non-mammalian *Angptl9* identified in this study was explored to understand its origin and evolution in vertebrates. Short-range gene linkage comparisons included human, chicken (*angptl9, angptl7*) or lizard (*angptl8,* as the chicken lacks a conserved gene environment), coelacanth, spotted gar and elephant shark and also two teleosts the tilapia and the zebrafish. The vertebrate neighbouring gene environment was retrieved from the Genomicus database [62] using the gene environment of human *angptl7* and *angptl8* and the spotted gar *angptl9* as the reference. The homologue genome regions in elephant shark and lamprey were characterized by querying their specific genome assemblies with the conserved flanking genes identified in teleosts and tetrapods. The identity of the cephalochordate genes was confirmed by their similarity with the human proteins.

### Sea bream skin regeneration challenge

Manipulation of animals was performed in compliance with international and national ethics guidelines for animal care and experimentation, under a “Group-I” license from the Portuguese Government Central Veterinary service to CCMAR and conducted by a certified investigator (DMP).

A stock of adult sea bream of the same age class (1 year) were purchased from a commercial supplier (CUPIMAR SA, Cádiz, Spain) and transferred to Ramalhete, the marine station of the Centre of Marine Sciences (CCMAR, University of the Algarve, Faro, Portugal). Fish were maintained in 1000 L tanks supplied with a continuous flow of aerated sea water at 18–20 °C, pH 7.8–8.1, 37 ppt salinity, >80% oxygen saturation and at a density of <5 kg · m^−3^. Fish were fed with a commercial feed (Excel; Skretting, Burgos, Spain) at 2% of the total kg of fish / tank twice daily.

For the skin regeneration challenge, adult sea bream (*N* = 48, length = 34 ± 1.3 cm) were divided randomly between five 500 L tanks (*N* = 8 per tank) supplied with a continuous flow of aerated seawater at 20 ± 2 °C and maintained under the conditions described above. For the skin regeneration challenge, fish were anesthetised with 2-phenoxyethanol in seawater (1:10,000; Sigma-Aldrich) and scales were removed from the left flank of the body by gently stroking the skin with forceps to minimise damage to the dermis. A group of fish (*N* = 8) were killed immediately (zero time) after scale removal and samples of intact skin (untouched right hand flank) and damaged skin (left hand flank) were snap frozen in liquid nitrogen and subsequently stored at −80 °C for molecular analysis or were fixed in 4% paraformaldehyde (4% PFA, pH 7.4) for histology. In this way, the same fish provided control and regenerating skin samples and they could be directly compared. To minimize undue stress to the fish the 5 tanks represented the different time points of the sample time series after scale removal: 6 h and day 1, 2, 3 and 4. Intact and regenerating skin samples (*N* = 8/ time point) were collected from fish anaesthetised in 2-phenoxyethanol (1:10,000, Sigma-Aldrich) and were weighed, length measured and a photograph taken. Fish were killed by decapitation and the skin below the dorsal fin on the left (regenerating skin) and right hand flank (intact skin) of the same fish was collected and a portion frozen in liquid nitrogen and the other portion fixed for histological examination.

### Skin histological and morphometric analysis

Intact and regenerating skin samples from sea bream (0 h, 6 h, 1, 2, 3 and 4 days after scale removal) were fixed in 4% PFA, decalcified overnight in 0.5 M ethylenediaminetetraacetic acid (EDTA, pH 8) and dehydrated in ethanol (70, 90 and 100%), saturated in xylene and impregnated and embedded in low melting point paraffin wax (Histosec, Merck). Serial 5 μm sections of skin were mounted on 3-aminopropyltriethoxysilane (APES) coated glass slides, dried overnight at 37 °C, cooled to room temperature and stored until required. Masson’s trichrome staining was used to distinguish between collagen rich and/or mineralized and non-mineralized tissue as previously described [8]. Stained sections were analysed using a microscope (Leica DM2000) coupled to a digital camera (Leica DFC480) linked to a computer for digital image analysis. Digital images were used to quantify the thickness of the epidermis, basement membrane and dermis as well as the number and diameter (20 vessels per section) of blood vessels in intact (*N* = 3, 1 section per fish) and regenerating (*N* = 3, 1 section per fish) skin using ImageJ v1.44o software [63].

### RNA extraction and cDNA synthesis

Total RNA was extracted from sea bream intact and regenerating skin using a Maxwell^®^ 16 MDx Instrument (Promega) and a Maxwell 16 Total RNA Purification Kit (Promega), according to the manufacturer’s instructions. The quality and integrity of total RNA was verified using a NanoDrop 1000 Spectrophotometer (Thermo Scientific). Purified total RNA (1–3 μg) was treated with 1.5 U DNAse (Ambion DNA-*free*™ kit) following the manufacturer’s instructions. DNA free total RNA (100 or 250 ng) was used for first strand cDNA synthesis in a 20 μl reaction volume containing 100 mM random hexamers (GE Healthcare, UK), 100 U of RevertAid™ Reverse Transcriptase (Fermentas) and 8 U of RiboLock™ RNase Inhibitor (Fermentas). cDNA was synthesized by incubating for 10 min at 20 °C, followed by 50 min at 42 °C and 5 min at 72 °C. The quality of cDNA was checked by PCR amplification of *rps18* with specific primers (Table 1) using the following cycle: 10 min at 95 °C, followed by 25 cycles of 95 °C for 30 s, 60 °C for 30 s and 72 °C for 30 s and a final 5 min at 72 °C. The PCR products were run on a 1% agarose gel to confirm amplicon size and the absence of contamination with genomic DNA.

**Table 1:** List of the primers used for gene expression analysis by quantitative real-time PCR in sea bream (*Sparus aurata*) skin

Symbol	Accession Number	Primer sequences (5’to 3’)	Amplicon (bp)	T (°C)	Efficiency (%)	R^2^
*angptl1b*		F: GCATGCAGGTCTACAGTCG R: CAAAGGCTCGGGTGTTGTC	135	58	96	0.99
*angptl2b*		F: TGCTGCACGAGATCATCAGGAA R: GTACTTGTGCTCGAGATCTTT	128	60	89	0.99
*angptl4a*		F: AGATACAGAAGGCTGATGCT R: CTGGTCGTTGTCTTGGTC	101	60	99	0.99
*angptl4b*		F: AAATAATGTCGACCGAAGAG R: CGAGTTACCACAGCTGTTG	128	60	81	0.99
*angptl7*		F: CAGTACGCTCAGGATCGAGATGG R: ATGGTGCTGAAGTTGGTGTTGTT	171	60	97	0.99
*vegfab*		F: ACGTCCAGCTATAACATTACAA R: CTTTCTTTAACCTACACTCA	115	58	90	0.99
*rps18*	AM490061	F: AGGGTGTTGGCAGACGTTAC R: CTTCTGCCTGTTGAGGAACC	164	60	88	0.99
*ß-actin*	X89920	F: CCCTGCCCCACGCCATCC R: TCTCGGCTGTGGTGGTGAAGG	94	60	92	0.99

Accession numbers, primer sequence, amplicon length (bp), annealing temperature (T °C) and qPCR efficiency (%) and R^2^ are indicated for each primer pair

*Abbreviations*: *F* forward, *R* reverse primer

### Quantitative expression analysis (qPCR)

The expression of *angptl1b*, *angptl2b*, *angptl3b*, *angptl4a*, *angptl4b*, *angptl7* and *angptl9b* was confirmed in sea bream skin and the abundance of the amplified transcripts were subsequently characterised in intact and regenerating skin by quantitative real-time PCR. Transcript specific primers were designed using the sea bream sequences as templates (Table 1) and qPCR was carried out in duplicate 10 μl reactions of 1× SsoFast-Evagreen Supermix (Biorad) containing cDNA (≈16.7 ng) and 300 nM of forward and reverse primers. Quantification was performed in a StepOnePlus thermocycler (Applied Biosystems, UK) using the standard-curve method (software StepOne™ Real-Time PCR Software v2.2) and the following program: 30 s at 95 °C, 45 cycles of 5 s at 95 °C and 15 s at 60 °C. A standard curve was included to permit the initial quantity of target template to be related to amplification cycle. A final melting curve was carried out between 60 and 95 °C and produced a single product dissociation curve for each gene. Relative expression (log2 (fold-change)) was estimated using the geometric mean of two-reference transcripts *rps18* and *ß-actin* that did not vary significantly (*p* > 0.05) between the control and regenerating skin samples.

### Statistical analysis

Significant changes in relative transcript expression in intact and regenerating skin during the wound healing process were assessed using a two-way ANOVA followed by a Fisher’s Least Significant Difference (LSD) post-test using the StatPlus:mac LE v5 2015 (AnalystSoft Inc., USA). Relative expression data are presented as mean ± standard error of the mean (SEM). Statistical significance was considered at *p* < 0.05. Significant differences in intact or regenerating skin at different time points during the experiment are annotated with different letters and significant differences between intact and regenerating skin at the same time point are annotated with an asterisk. Correlation analysis was performed with GraphPad Prism version 6.00 for Macintosh, (GraphPad Software, La Jolla California USA) to associate patterns of gene expression with key morphological events (epidermis closure, basement membrane and dermis thickening; and development of and diameter of blood vessels) during the initial phases of piscine skin regeneration.

## Results

### Angptls in fish and other metazoans

**Fig. 1:**
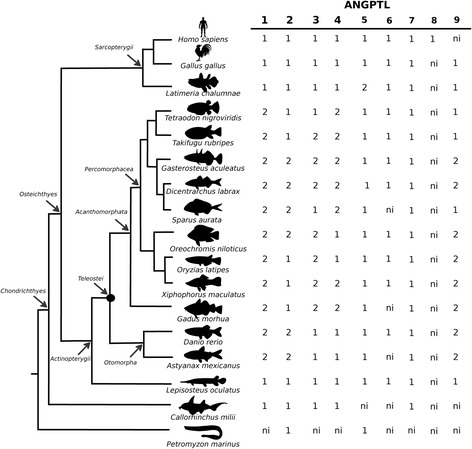
*Angptl* gene family members in fish. The number of predicted genes is indicated. The chicken and human genes are also indicated to allow comparisons with the fish homologues. “●“ represents the Teleost Specific Genome Duplication (TSGD). *ni*: not identified

Sequence homologues of human ANGPTL family members were identified in several fish. A previously undescribed member of this family was identified in fish genomes and also in *Xenopus*, lizard and chicken genomes and was designated *angptl9*. The new ANGPTL family member was absent from mammalian genomes. In contrast, orthologues of human *ANGPTL8* were absent from fish and other non-mammalian vertebrate genomes (Fig. 1 and Additional file 1: Table S1). Ten teleost fish genomes were analysed and the total *angptl* gene number retrieved per genome varied from 10 to 13 depending on retention or not of duplicate gene copies of *angptl1*, *angptl2*, *angptl3* and *angptl4* and the new *angptl9* gene identified in this study. Duplicates of human *ANGPTL1* gene homologues were identified in all teleost genomes analysed but the persistence of paralogues for the other family members was species-dependent and it was not possible in some species to establish if the full complement of genes was present due to the incompleteness of their genome assemblies.

In the genome of the spotted gar (*Lepisosteus oculatus*), which diverged prior to the teleost radiation, a single *angptl* gene copy was found. The lobe-finned coelacanth, a fish basal to tetrapods, had a similar gene repertoire to the spotted gar with the exception of the duplicate *angptl5* genes. In the cartilaginous fish the elephant shark (*Callorhinchus milii*) and the little skate (*Leucoraja erinacea*), 5 and 4 *angptl* genes, respectively were retrieved and orthologues of the teleost *angptl5*, *angptl6* and *angptl9* remain to be identified (Fig. 1 and Additional file 1: Table S1). Searches in the jawless fish, the marine lamprey (*Petromyzon marinus*), recovered putative *angptl2* and *angptl5* genes but the incomplete nature of its genome assembly meant that the existence of other family members was not established (Fig. 1 and Additional file 1: Table S1). In the gilthead sea bream (*Sparus aurata*) that does not have a sequenced genome, 10 *angptl* transcripts were retrieved but the orthologues of the teleost *angptl3a* and *angptl6* were not identified (Fig. 1 and Additional file 1: Table S1).

Terrestrial vertebrates including, the amphibian (*Xenopus tropicalis*), the anole lizard (*Anolis carolinensis*), the chicken (*Gallus gallus*), the opossum (*Monodelphis domestica*) and the mouse *(Mus musculus*) had a similar gene repertoire to human but in non-mammalian genomes an orthologue of the fish *angptl9* gene also existed. Orthologues of human *ANGPTL8* were only identified in mammals and were absent from other vertebrates.

In the cephalochordate (*Branchiostoma floridae*) at least 5 putative *angptl-like* genes were identified (Additional file 1: Table S1) indicating that this gene family is ancient and arose prior to the vertebrate radiation. Data mining of other early deuterostome genomes failed to retrieve annotated genes although predicted transcripts orthologous to human *ANGPTL1* were identified in a urochordate, the sea squirt (*Ciona intestinalis*, XM_002126240), in an echinoderm, the sea urchin (*Strongylocentrotus purpuratus*, XM_781185, XM_003727342), and in a hemichordate, the acorn worm (*Saccoglossus kowalevskii*, XM_002739547 and XM_006819919). An orthologue of human *ANGPTL2* (XM_001178311) was also identified in the sea urchin, indicating that different members of the Angptl family are present in non-vertebrate genomes. However, their deduced transcripts are highly divergent in sequence (<20% aa sequence similarity) and length (generally much longer) with the putative human homologues and were not considered for further analysis. In protostomes sequence hits for proteins related to the vertebrate ANGPTLs such as tenascins, ficolins, fibrinogen and others were also obtained but were not explored in this study.

### Phylogeny of the fish angptls

**Fig. 2:**
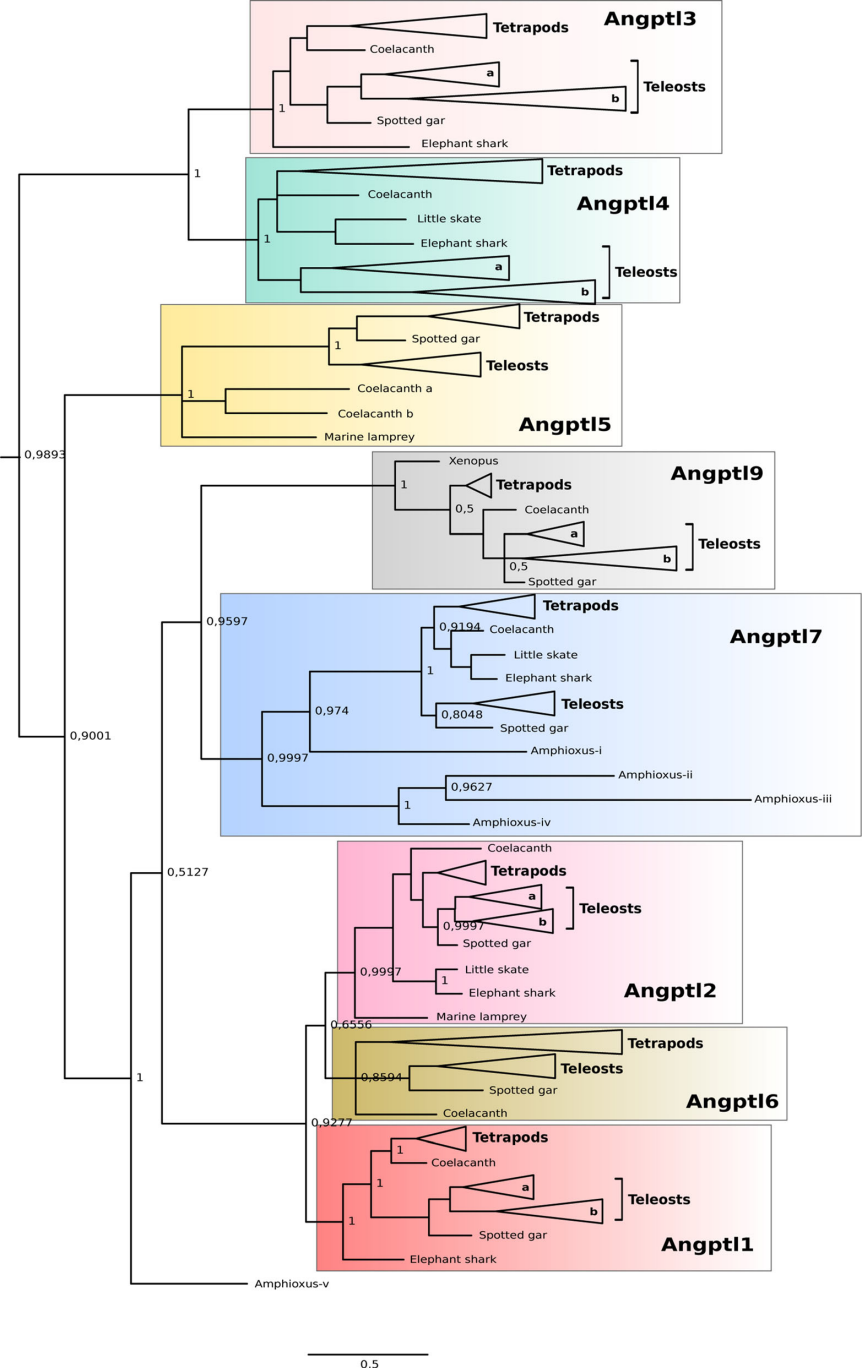
Phylogenetic tree of the fish *Angptl* with the tetrapod and cephalochordate homologues. Tree was constructed with the Bayesian interference (BI) built in MrBayes 3.2 and branch support values (*posterior probability values*) are shown only for the major protein family clades. The *Angptl* family clusters are boxed with different colours. The phylogenetic tree is compacted and was generated from the original tree available in Additional file 2: Figure S1. The tree was rooted using the deuterostome Angpt clade (*Angpt1, Angpt2 and Angpt4*) but details are not show in the figure to facilitate interpretation. For the same reason, the detailed presentation of the teleost and tetrapod members of each family group have been collapsed. A tree with similar topology was obtained using the Maximum-likelihood algorithm and 100 bootstrap replicates (Additional file 3: Figure S2). ANGPTL8 was not included in the phylogenetic analysis as it is an atypical family member and lacks the FReD domain

Phylogenetic analysis of the vertebrate and cephalochordate Angptl family revealed that the genes shared a common origin and that the family members emerged early during the deuterostome radiation (Fig. 2 and Additional file 2: Figure S1 and Additional file 3: Figure S2). According to the tree topology, four main protein clusters that contain distinct members of the Angptl family exist: the Angptl1-2-6 cluster (Angptl1, Angptl2, Angptl6), the Angptl3-4 cluster (Angptl3, Angptl4), the Angptl5 cluster (Angptl5) and the Angptl7-9 cluster (Angptl7, Angptl9). According to the tree topology, the Angptl3-4 cluster diverged early after the gene duplication event that gave rise to the ancestral gene from which the Angptl1-2-6, Angptl5 and Angptl7-9 subsequently emerged. This suggests that the ancestral *Angptl* gene duplicated prior to the radiation of the vertebrates and that the family members arose from different ancestral genes.

In teleosts, duplicate copies of *angptl1*, *angptl2*, *angptl3, angptl4* and *angptl9* arose from the whole genome duplication event reported in this lineage [64]. The teleost *angptl* gene duplicates are differentiated using the letters *a* and *b* and the gene environment of paralogue *a* shares the greatest conservation with the homologue chromosome regions in human and spotted gar [62]. In other non-teleost fish, single gene family members exist with the exception of *Angptl5* that is duplicated in the coelacanth genome.

The five cephalochordate *angptl-like* genes clustered with the vertebrate Angptls (Fig. 2). Four of which group within the vertebrate Angptl7 clade and three of which (Amphioxus_ii, Amphioxus_iii and Amphioxus_iv) seem to have arisen due to a species-specific gene duplication. The fifth Angptl (Amphioxus_v) sits in the phylogenetic tree prior to the emergence of the vertebrate Angptl1-2-6 and Angptl7-9 clades (Fig. 2). The existence of other *angptl* genes in amphioxus was not established but they are likely to exist and were not identified due to the incompleteness of the genome assembly.

### Sequence conservation of the fish angptls with human and cephalochordate

**Fig. 3:**
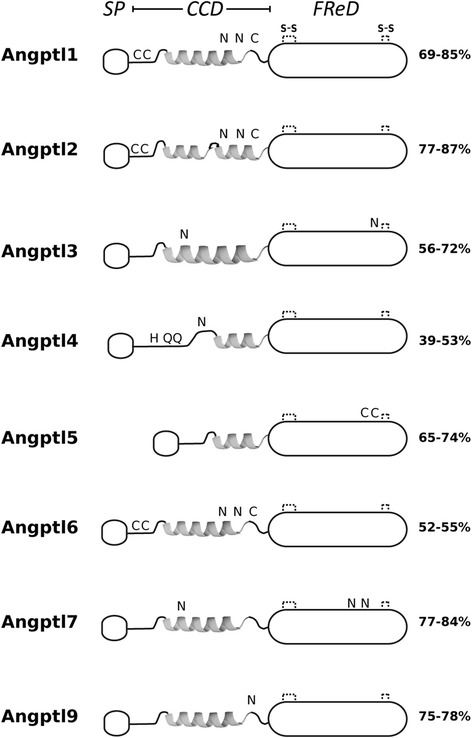
Schematic representations of the deduced structure and conserved consensus motifs of the fish and human ANGPTLs. The signal peptide region (*SP, small open box*), the coiled-coil domain (*CCD, helix*) and the highly conserved fibrinogen-related domain (*FReD, long open box*) are annotated and to facilitate visualization protein structures were aligned using the FReD motif. The four conserved cysteine residues within the FReD motif potentially involved in the establishment of two intramolecular disulphide bonds are represented and indicated by “S-S” in the Angptl1 protein structure and their positions were obtained from Uniprot annotation. Other vertebrate conserved cysteine residues are represented by “C” and predicted N-glycosylation (N-x-T/S) motifs are annotated with “N”. Across fish, the amino acid residues that regulate the activity of the human ANGPTL4 (His^46^, Gln^50^ and Gln^53^) [66] are also conserved. The figure is not drawn to scale and the percent of amino acid sequence similarity between the fish and the human orthologues is indicated with the exception of Angptl9 that considers the similarity across fish. The ANGPTL 8 is not represented, as it is only present in mammals. The complete alignments of the human and fish Angptl proteins are available in Additional file 4: Figure S3a–d. * The sequence similarity of the coelacanth duplicates was not considered, as their sequence was highly divergent

Amino acid (aa) sequence alignment of the fish ANGPTLs with the human orthologues revealed that they are highly conserved and protein domains and sequence motifs are shared by teleost and tetrapod sequences (Fig. 3 and Additional file 4: Figure S3a–d). This includes an N-terminal coiled-coil domain (CCD) and a highly conserved fibrinogen-related domain (FReD) in the C-terminal region that is also present in ANGPT proteins [23]. Within the FReD motif, four highly conserved cysteine residues predicted to establish two intramolecular disulphide bonds were conserved in human and fish, however their importance in protein structure and function still remain to be established [65] (Fig. 3). Human and fish Angptl1, 2 and 7 were the most highly conserved members and their deduced mature protein sequence share at least 69, 77 and 77% aa sequence similarity, respectively (Additional file 5: Table S2). The deduced sequence of Angptl9 was also highly conserved and shared 75–78% aa similarity between fish and non-mammalian tetrapods. The most divergent forms of Angptl were Angptl4 and Angptl6 that shared a maximum of 53 and 55% aa sequence similarity between fish and human, respectively (Additional file 5: Table S2).

Although overall all the vertebrate Angptl family members shared relatively high sequence conservation some specific amino acid sequences that have been linked to protein processing or protein structural configuration/function were also common across fish and human (Fig. 3 and Additional file 4: Figure S3a–d). This included the three cysteine residues and two glycosylation sites within the CCD of Angptl1, Angptl2 and Angptl6; an N-glycosylation site (CCD motif) and a N-glycosylation site (FReD) in Angptl3; an N-glycosylation site in the CCD of vertebrate Angptl4 and the amino acids His^46^, Gln^50^ and Gln^53^ that are important for the regulation of lipoprotein lipase (LPL) in humans and the elevation of plasma triglyceride levels in mice [66]; the vertebrate Angptl5 contained two conserved cysteine residues that may form an extra disulphide bridge within the FReD domain; and three N-glycosylation sites, one within the CCD and two in the FReD domains were conserved across the vertebrate Angptl7. A unique N-glycosylation site within the CCD was predicted in the deduced protein of Angptl9 from the teleosts and the spotted gar.

The deduced cephalochordate Angptl-like (i to iv) proteins shared the highest sequence similarity (29–37% aa) with vertebrate Angptl7 and within the deduced lamprey proteins the FReD domain was the most highly conserved region (Additional file 6: Figure S4).

### Neighbouring gene analysis

**Fig. 4:**
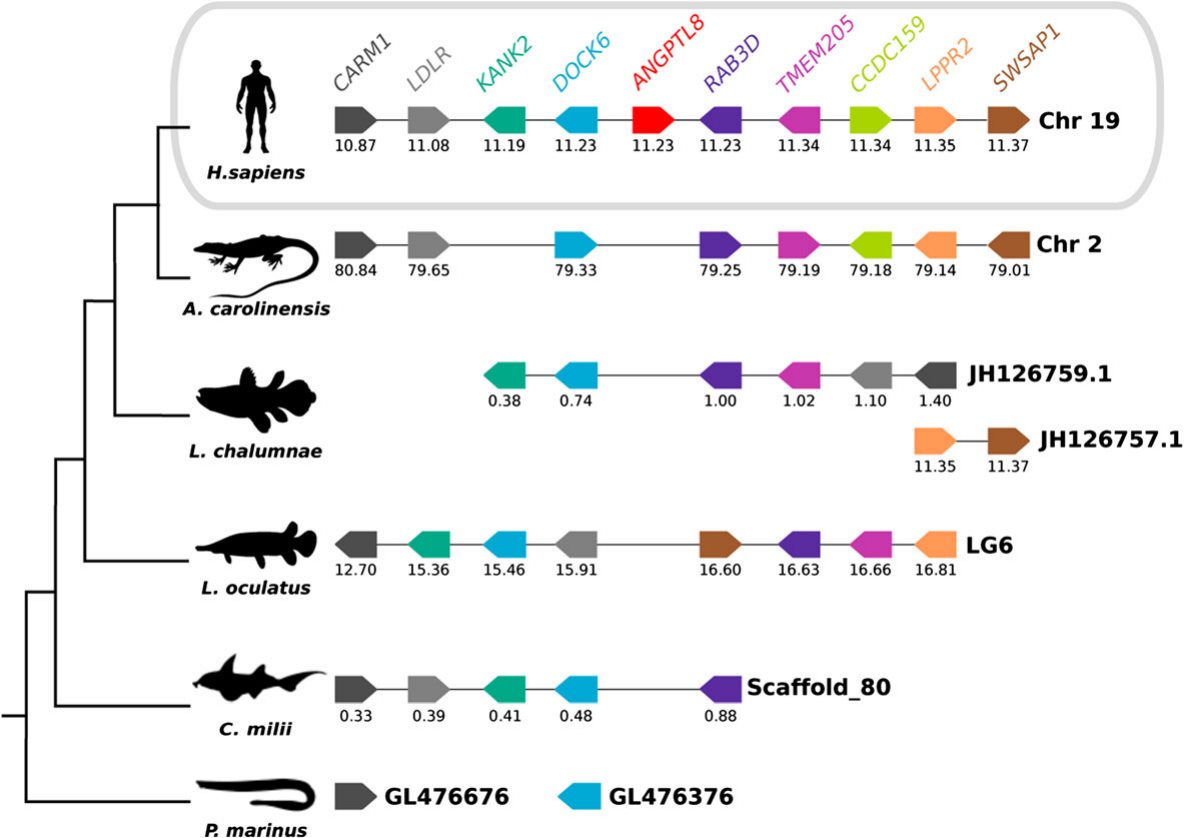
Comparison of the homologous genome regions harbouring the human *ANGPTL8* with the fish and lizard. The gene environment of the human *ANGPTL8* gene was characterised and was used to identify homologous genes in the Anole lizard and several fish: the coelacanth (*lobed-finned fish*), the spotted gar (*ray-finned fish*), the elephant shark (*cartilaginous fish*) and the marine lamprey (*jawless fish*) genomes. *Horizontal lines* represent the chromosome fragments; *arrow boxes* indicate genes and the arrowhead points in the direction of the predicted gene transcription. Only genes that were conserved across species are represented. Gene names are indicated according to the human annotation and the same colour is used for gene homologues and they are presented according to their order in the chromosome. The size of the genome fragments analysed and the predicted location of the genes in the chromosomes are indicated in Megabase pairs. Gene names and symbols are: Coactivator-Associated Arginine Methyltransferase 1 (*CARM1*), Low Density Lipoprotein Receptor (*LDLR*), KN Motif and Ankyrin Repeat Domains 2 (*KANK2*), Dedicator Of Cytokinesis 6 (*DOCK6*), Member RAS Oncogene Family (*RAB3D*), Transmembrane Protein 205 (*TMEM205*), Coiled-Coil Domain Containing 159 (*CCDC159*), Lipid Phosphate Phosphatase-Related Protein Type 2 (*LPPR2*) and SWIM-Type Zinc Finger 7 Associated Protein 1 (*SWSAP1*)

**Fig. 5:**
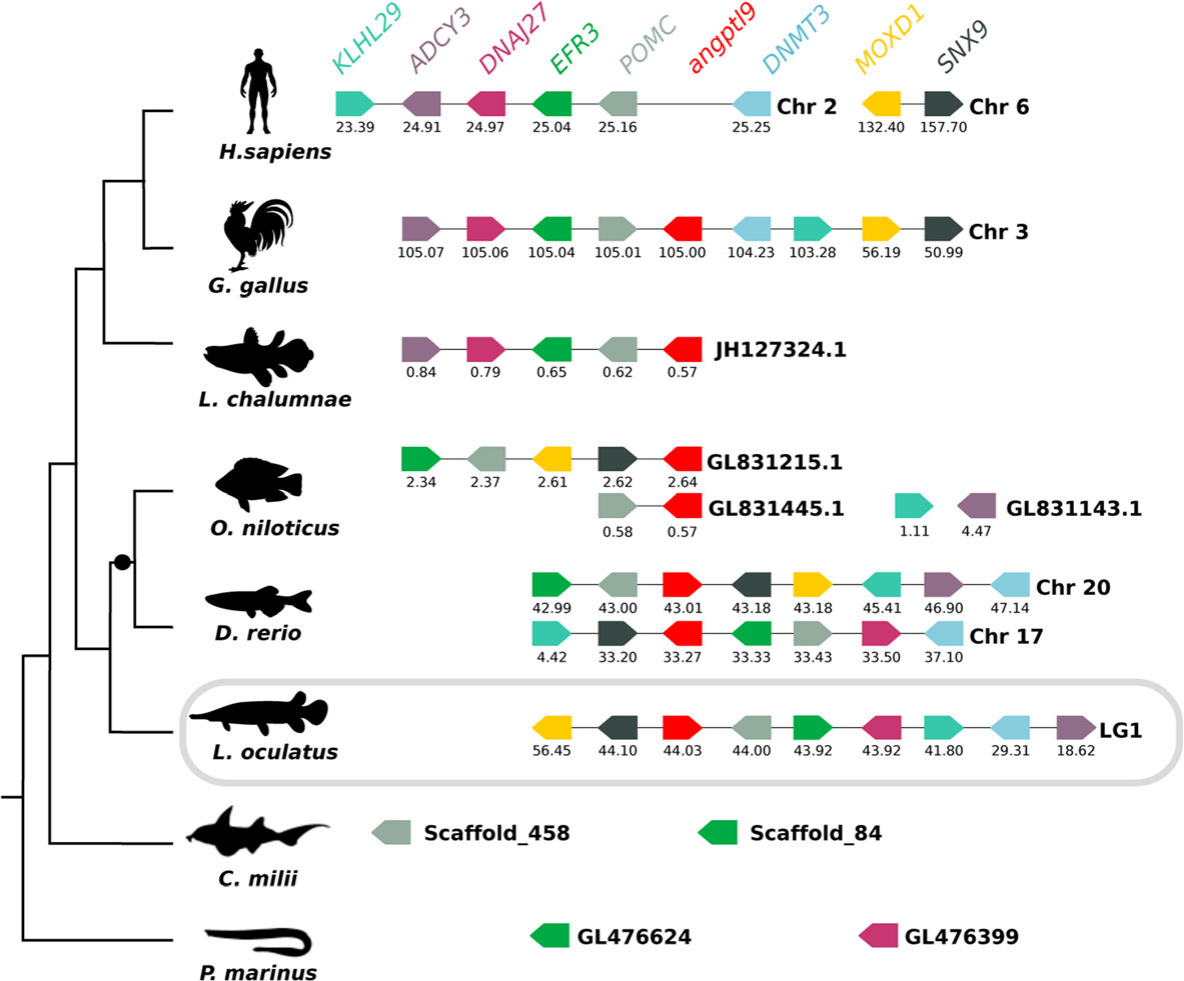
Comparison of the spotted gar genome region harbouring *angptl9* with other fish, chicken and human. The gene environment of the spotted gar *angptl9* gene was characterised and was used to identify homologous genome regions in the teleost (*zebrafish and tilapia*), the coelacanth (*lobe-finned fish*), the elephant shark (*cartilaginous fish*), marine lamprey (*jawless fish*), chicken and human. *Horizontal lines* represent the chromosome fragments; *arrow boxes* indicate genes and the arrowhead points to the orientation of the predicted gene transcription. Only genes that were conserved across species are represented. Gene symbols are indicated and homologue genes are represented by the same colour and they are represented according to their order in the chromosome. The size of the genome fragments analysed and the location of the gene in the chromosome are indicated in Megabase pairs. “●”: Teleost Specific Genome Duplication (TSGD). Gene names and symbols are: Kelch-like family member 29 (*KLHL29*), Adenylate cyclase 3 (*ADCY3*), DnaJ (*HSP40*) homolog, subfamily C member 27 (*DNAJC27*), EFR3 (*EFR3*), Proopiomelanocortin (*POMC*), DNA (cytosine-5)-methyltransferase 3 (*DNMT3*), Monooxygenase, DBH-like 1 (*MOXD1*) and Sorting nexin 9 (*SNX9*)

**Fig. 6:**
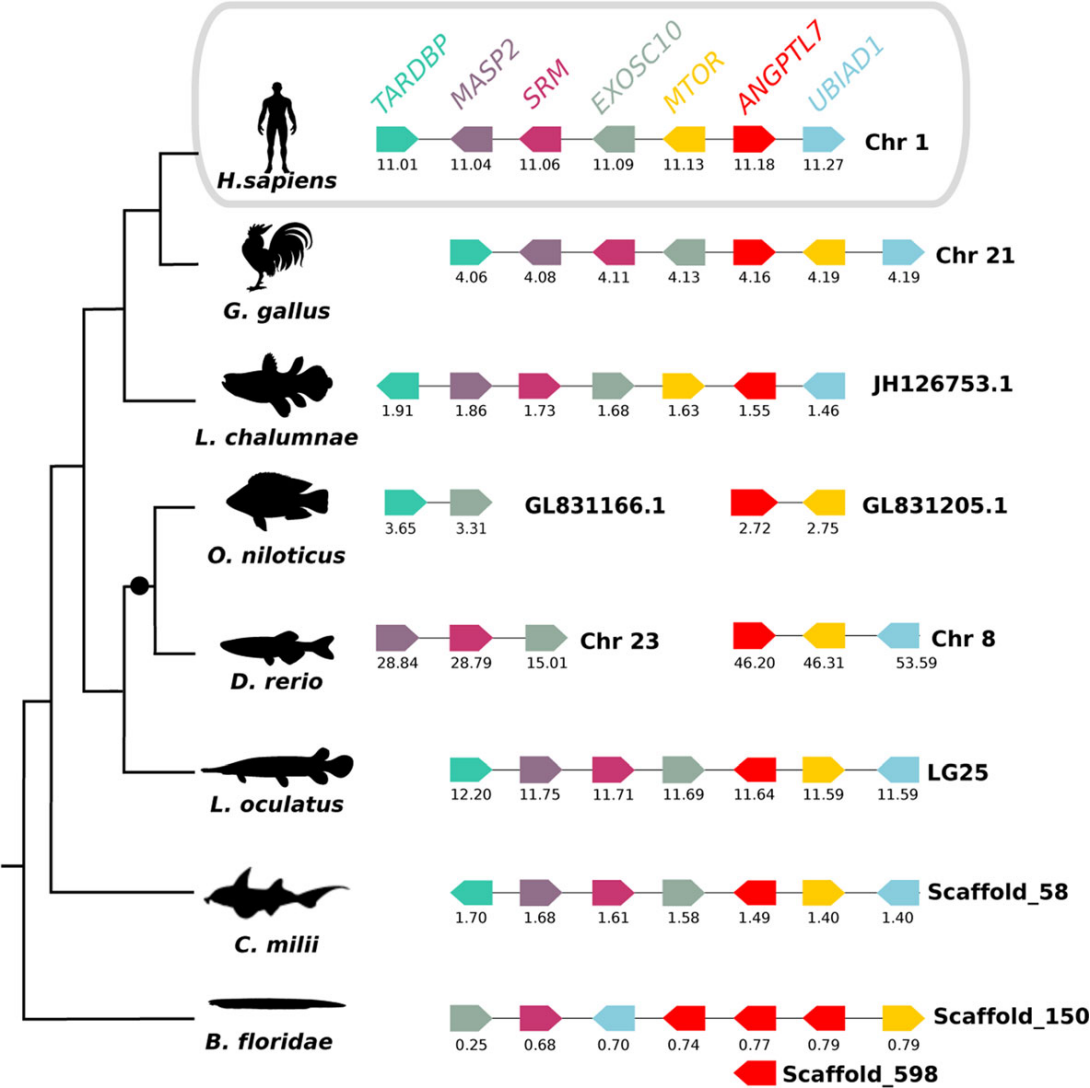
Comparison of the vertebrate and cephalochordate *angptl7* homologous genome regions. The vertebrate *angptl7* conserved gene environment was characterised using the human genome region as reference and was used to identify a homologous region in the amphioxus genome region that house the cephalochordate *angptl7-like* genes. *Horizontal lines* represent chromosome fragments and its names and genome fragments are indicated at the right side; *coloured block arrows* represent genes according to the order in the chromosome and the arrowhead points to the predicted gene transcription. The location of the gene in the chromosome is indicated below each arrow, in Megabase pairs. “●”: Teleost Specific Genome Duplication (TSGD). Gene names and gene symbols are: TAR DNA binding protein (*TARDBP*), Mannan-binding lectin serine protease 2 (*MASP2*), Spermidine synthase (*SRM*), Exosome component 10 (*EXOSC10*), Mechanistic target of rapamicin (serine/threonine kinase) (*MTOR*) and UbiA prenyltransferase domain containing 1 (*UBIAD1*)

To better understand the evolution of the *Angptl* gene family during the vertebrate radiation, the neighbouring gene environment of mammalian *ANGPTL 8* (Fig. 4) and non-mammalian *Angptl 9* (Fig. 5) were compared between fish and tetrapods. The gene environment of the cephalochordate *Angptl-like* genes that cluster with the vertebrate Angptl7 clade was also characterised (Fig. 6). In human, the *ANGPTL8* gene mapped to chromosome 19 and orthologues of the flanking genes were found in other vertebrate genomes. A chromosome region with a similar gene repertoire to that flanking human *ANGPTL8* was found in the lizard and in the fish (coelacanth, spotted gar and elephant shark, Fig. 4) even though the *ANGPTL8* gene was absent from their genomes. Of the nine genes that flank human *ANGPTL8*, eight retain linkage in chromosome 2 of the lizard and in chromosome LG6 of the spotted gar genome suggesting the loss of this gene in these species is potentially a consequence of lineage specific gene deletions (Fig. 4).

In the spotted gar, the *angptl9* gene maps to LG1 and in the chicken to chromosome 3 and eight genes in linkage were identified. In the human genome a chromosome region was identified that was homologous to the gene environment flanking the fish *angptl9* gene even though the gene has been lost from mammalian genomes (Fig. 5). The genes that flank the *angptl9* gene in fish and in chicken are shared between two human chromosomes (chromosome 2 and 6) indicating reorganisation of this genome region during the radiation of mammals. Characterisation of the neighbouring gene environment of the duplicate teleost *angptl9* genes revealed that they map to genome regions that share a similar gene complement confirming that they emerged from the teleost genome tetraploidization.

The gene environment of *Angptl7* in fish (elephant shark, spotted gar, teleost and coelacanth) and tetrapods revealed similarity with cephalochordate scaffold_150 that houses amphioxus *Angptl-like*_ii, iii and iv genes and suggests that vertebrate and cephalochordate *angptl7* shared a common ancestral origin (Fig. 6). The neighbourhood of amphioxus *angptl-like*_i that maps to scaffold_598 shared no gene linkage with any of the vertebrate chromosomes/scaffolds containing *Angptl* genes and its location may be the result of gene duplication and subsequent translocation.

### Morphological and morphometric evaluation of sea bream intact and regenerating skin

**Fig. 7:**
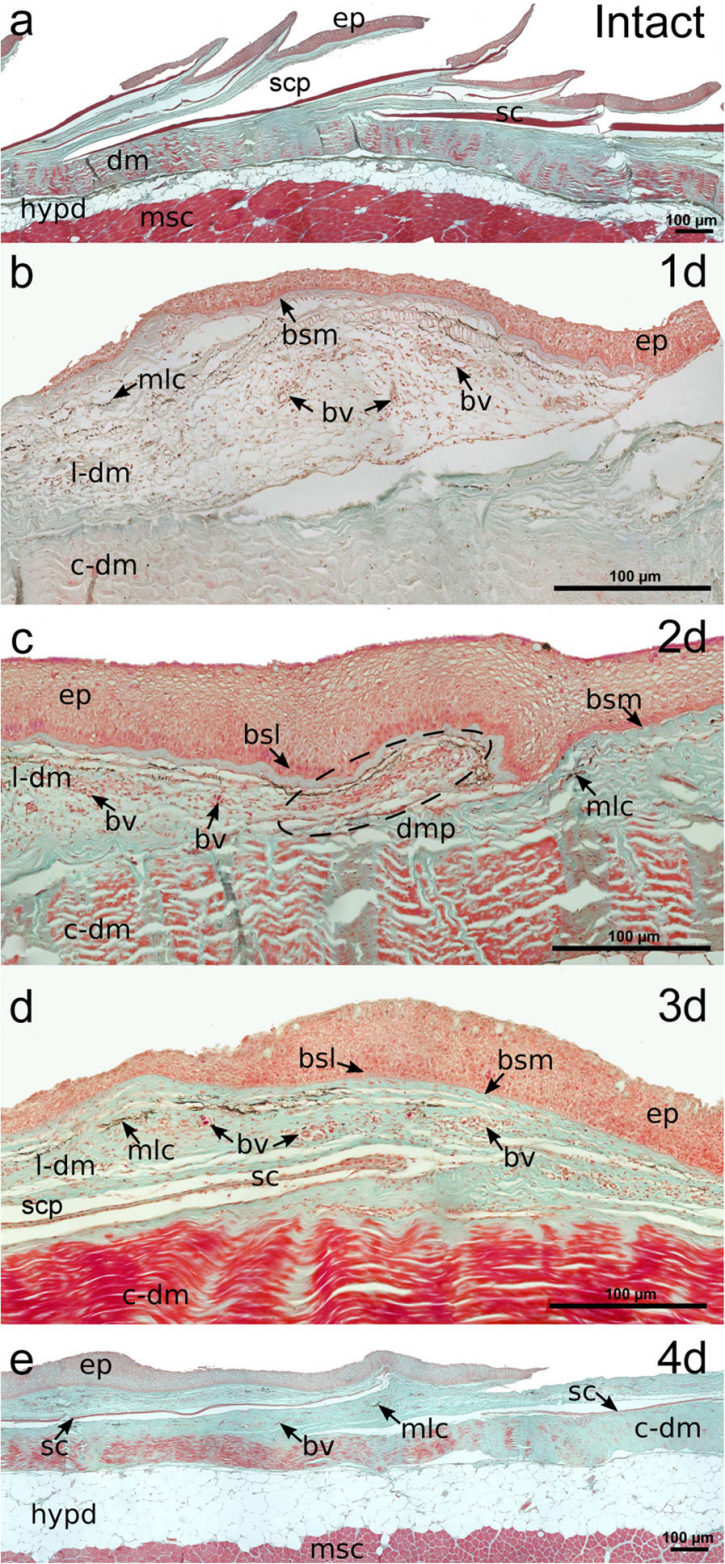
Morphological evaluation of sea bream intact and regenerating skin (*1, 2, 3 and 4 days after wounding*) stained with Masson’s trichrome. The posterior region of the scale is orientated to the right. Connective tissue is stained green and mineralized and collagen-rich tissues are stained bright red. **a** Intact skin before scale removal; **b**–**e** Regenerating skin at 1, 2, 3 and 4 days after wounding, respectively. Ep: epidermis; dm: demis; dm-l: loose dermis; dm-c: compact dermis; hyd: hypodermis; sc: scale: scp: scale pocket; msc: muscle; bsm: basement membrane; bsl: basal layer; dmp: dermal papilla; mlc: melanocytes; blv: blood vessel

Longitudinal transverse sections of intact and regenerating sea bream skin samples were used to characterize the ontogeny of tissue regeneration after scale removal (Fig. 7). The three typical layers, the epidermis, dermis and hypodermis were observed in histological sections of intact sea bream skin. The scales sat in individual scale pockets, inserted in the dermis and several layers of mineralized collagen were visible (Fig. 7a). The removal of scales damaged the epidermis, dermis and scale pocket and 1 day after scale removal (Fig. 7b) the torn edges of the ruptured epidermis although still attached to the skin left the dermis and scale pocket exposed directly to the aquatic milieu. Blood vessels were observed in the loose dermis but not in the compact dermis. Fast re-epithelialization of the epidermis occurred and 2 days after scale removal a new epidermis covered the dermis (Fig. 7c). A continuous basal layer and basal membrane were observed and formed an interface between the epidermis and the loose dermis. The scale papilla was also evident in the loose dermis 2 days after scale removal and delineated the location of the future scale pocket and new scale. Establishment of the external barrier was completed 2 days after scale removal (Fig. 7d) and numerous blood vessels were observed in the loose dermis. A thin layer of non-mineralized tissue was visible inside the scale pocket 3 days after scale removal and corresponded to the forming scale. By day 4 after scale removal (Fig. 7e) the structure of the regenerating skin already resembled that of the intact skin, although the mineralized scale was very thin and still did not correspond in thickness or size to the ontogenetic scale.

**Fig. 8:**
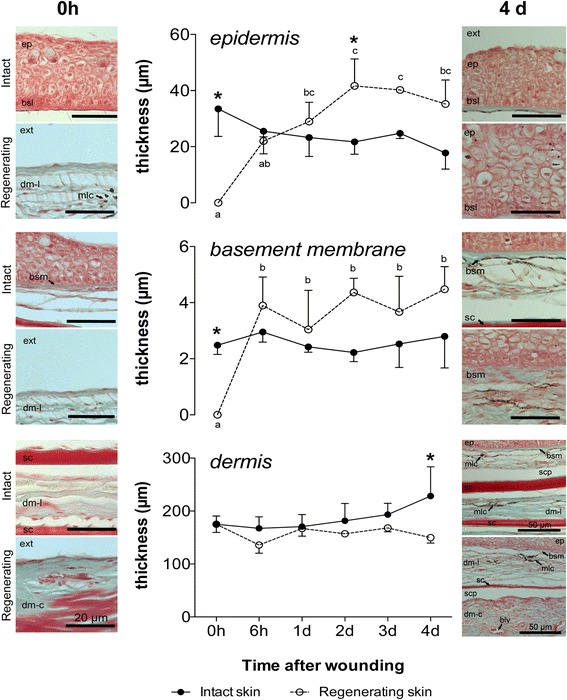
Morphometric evaluation of sea bream skin during wound healing after scale removal. The thickness of the epidermis, basement membrane and dermis during sea bream skin recovery after scale removal is represented. Each value represents the mean ± SEM (*N* = 3). Statistical significance between groups was assessed using a two-way ANOVA followed by Fisher’s Least Significance Difference (LSD) post-test. Statistical significances were considered at *p* < 0.05 and differences in intact and regenerating skin during the experimental trial are annotated with different letters and comparisons between intact and regenerating skin at the same time point are signalled with an asterisk. A histological image of the skin layers at the start (*0 h*) and end (*4 days after wounding, daw*) of the experimental trial is represented beside each graph in order to illustrate the induced tissue aggression and the respective recovery with time. Ep: epidermis; bsl: basal layer; bsm: basement membrane; dm-l: loose dermis; dm-c: compact dermis; sc: scale; scp: scale pocket; mlc: melanocytes; blc: blood vessel; ext: exterior. Scale bars: 20 μm (*scale bars alone*) and 50 μm

Morphometric evaluation of the sea bream skin (Fig. 8) revealed the most dramatic changes in the regenerating skin where a marked increase in the thickness of the basement membrane (*p* = 0.04) from 6 h onwards and this was positively correlated (*r* = 0.487, *p* = 0.003, Additional file 7: Table S3a) with the progressive increase in the thickness of the epidermis (*p* = 0.04) from 1 day onwards compared to time 0. No changes in the thickness of the dermis were observed during the experiment (*p* > 0.05). In the intact skin the thickness of the epidermis (22.87 ± 1.151 μm), basement membrane (2.53 ± 0.121 μm) and dermis (185.4 ± 9.431 μm) remained constant throughout the healing period.

**Fig. 9:**
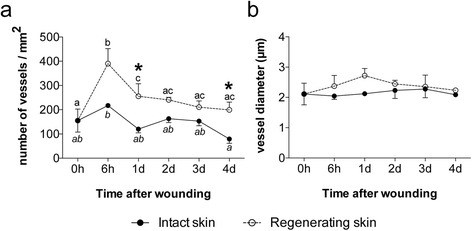
**a** Number of blood vessels and **b** blood vessel diameter during skin wound healing in sea bream. Each value represents the mean ± SEM (*N* = 3). Statistical significance between groups was assessed using two-way ANOVA followed by Fisher’s Least Significance Difference (LSD) post-test. Statistical significance was considered at *p* < 0.05 and differences in intact and regenerating skin during the experimental trial are annotated with different letters and comparisons between intact and regenerating skin at the same time point are signalled with an asterisk

In order to assess the recovery of the vascular system in regenerating skin during the healing process the number and diameter of the blood vessels in the different periods analysed were determined (Fig. 9). An increase in the number of blood vessels was observed at 6 h after wounding in the regenerating skin (*p* = 0.026) compared to time 0 (Fig. 9a). This was subsequently followed by a decrease in the number of blood vessels until day 2 after wounding when similar numbers to that observed at time 0 were found, thereafter their number remained relatively constant (*p* < 0.05). Variation in the number of blood vessels was positively correlated with the increase in the thickness of the epidermis (*r* = 0.421, *p* = 0.012) and basement membrane (*r* = 0.385, *p* = 0.023) (Additional file 7: Table S3a). Analysis of the blood vessel diameter (Fig. 9b) revealed that an increase in diameter was progressively observed during the first 24 h post wounding in regenerating skin. Blood vessel diameter was relatively constant in intact skin (*p* > 0.05). This revealed that the increase in blood vessel number 6 h after wounding in the damaged skin was not the consequence of improved detection due to vasodilation and suggests that new blood vessels were formed.

### Expression of *angptl* family members during sea bream skin regeneration

Analysis of *angptl* transcript distribution in fish by ESTs in NCBI revealed that they have a widespread tissue distribution (Additional file 8: Table S4). The results of *in silico* analysis (EST, sea bass skin transcriptome and sea bream skin microarray) indicated that *angptl2b*, *angptl3b*, *angptl4a*, *angptl4b*, *angptl7* and *angptl9b* transcripts are expressed in fish skin (Table 2 and Additional file 8: Table S4). Verification by qPCR using cDNA from sea bream skin confirmed the presence of *angptl1b*, *angptl2b*, *angptl4a*, *angptl4b* and *angptl7* transcripts but not *angptl3b* and *angptl9b* and they were excluded from further analysis.

**Table 2:** Digital expression analysis of *angptls* transcripts in the teleost skin

*Symbol*	Teleost EST	Sea bass transcriptome	Sea bream microarray
*angptl1a*	ni	ni	ni
*angptl1b*	ni	ni	ni
*angptl2a*	ni	ni	ni
*angptl2b*	ni	1050028/1053154	ni
*angptl3a*	ni	ni	ni
*angptl3b*	ni	ni	SAPD06471_1/
*angptl4a*	ni	1054686	SAPD06461_1/ SAPD06461_2
*angptl4b*	GH688340	1076279/1094540	ni
*angptl5*	ni	ni	ni
*angptl6*	ni	ni	ni
*angptl7*	AM979347/DT055381	1091792	SAPD09662_2
*angptl9a*	ni	ni	ni
*angptl9b*	ni	1099452	ni

Searches were performed against the teleost NCBI database, sea bass skin transcriptome (Patricia Pinto, *personal communication*) and sea bream skin scale microarray probes [8] ni: not identified

**Fig. 10:**
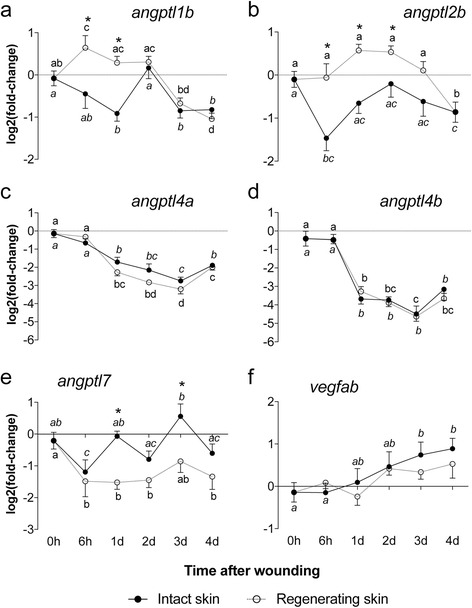
Relative expression of the sea bream *angptl1b*, *angptl2b*, *angptl4a*, *angptl4b*, *angptl7* and *vegfab* in intact and regenerating skin. Expression levels were obtained by qPCR and each value, that represents the mean ± SEM (*N* = 6) of the relative expression (log2 (*fold-change*)), was estimated using the geometric mean of *rps18* and *ß-actin* in intact and regenerating skin at time 0 h, 6 h and days 1, 2, 3 and 4 after wounding. Statistically significant differences between groups was assessed using two-way ANOVA followed by the Fisher’s Least Significant Difference (LSD) post-test. Statistical significances were considered at *p* < 0.05 and differences in intact and regenerating skin during the experimental trial are annotated with different letters and comparisons between intact and regenerating skin at the same time point are signalled with an asterisk

The abundance of *angptl1b*, *angptl2b*, *angptl4a*, *angptl4b* and *angptl7* transcripts was evaluated during sea bream skin regeneration along with *vegfab*, a mediator of vascular development in zebrafish [67] (Fig. 10). The transcript abundance of *angptl1b* and *angptl2b* was significantly increased (6 h and 1 day, *p* < 0.05) at initial stages of skin regeneration compared to the undamaged skin (from the other flank of the same fish) and subsequently decreased significantly (3 days and 4 days, *p* < 0.05). The abundance of *angptl1b* and *angptl2b* transcripts during the sea bream skin regeneration was correlated (*r* = 0.559, *p* < 0.001, Additional file 7: Table S3b).

In contrast, *angptl7* was significantly down-regulated from 6 h to 4 days (*p* < 0.05) in regenerating skin relative to time 0. The transcription of *angptl4a* and *angptl4b* was similar and significantly (*p* < 0.05) down-regulated from 1 day to 4 days in both intact and regenerating skin relative to time 0. The change in transcript abundance over time of *angptl4a* and *angptl4b* were highly correlated (*r* = 0.884, *p* < 0.001; Additional file 7: Table S3b) and no significant differences in transcript abundance existed between intact and regenerating skin.

The expression of *angptl1b* (Fig. 10a) in regenerating skin relative to skin at time 0 was significantly increased at 6 h (*p* = 0.01028) up until day 2 and subsequently decreased at day 3 and 4 when it was significantly lower (*p* = 0.00083) than at the start of the experiment. In intact skin taken from the undamaged flank of sea bream, *angptl1b* decreased significantly from day 1 (*p* = 0.00353), 3 (*p* = 0.01061) to 4 (*p* = 0.0087) relative to the start of the experiment (time 0). Pairwise comparisons of *angptl1b* transcript abundance in intact and regenerating skin at each time point revealed significant up-regulation in regenerating skin at 6 h (*p* = 0.00053) and 1 day (*p* = 0.00015) after scale removal.

*Angptl2b* (Fig. 10b) transcripts in regenerating skin were significantly up-regulated (*p* < 0.05) at days 1, 2 and 3 and then strongly and significantly down-regulated at day 4 (*p* = 0.01908) compared to skin at time 0. In the intact skin *angptl2* transcripts were significantly decreased at 6 h (*p* = 0.00005) and day 4 (*p* = 0.01823) relative to time 0. In contrast, pairwise comparisons of intact and regenerating skin at each time point revealed that *angptl2b* transcripts were significantly up-regulated in the regenerating skin relative to intact skin at 6 h (*p* = 0.00069) and 1 day (*p* = 0.00011) after scale removal (time 0).

*Angptl4a* and *angptl4b* (Fig. 10c and d) transcripts had a similar pattern of expression and their abundance decreased progressively after scale removal and were significantly down-regulated (*p* < 0.001) 1 day after scale removal in both intact and regenerating skin. Pairwise comparisons of intact and regenerating skin in the same individual at each time point did not reveal any significant differences in *angptl4a* and *angptl4b* transcript abundance.

Expression of *angptl7* was variable in both intact and regenerating skin samples over the 4 days of the experiment (Fig. 10e). In regenerating skin, *angptl7* was significantly down-regulated (*p* < 0.001) at 6 h and on days 1, 2 and 4 after scale removal relative to time 0. In intact skin, a*ngptl7* transcripts were significantly down-regulated (*p* = 0.002) 6 h after the start of the experiment, then significantly up-regulated at day 1 (*p* < 0.001) and day 3 (*p* < 0.001) relative to 6 h. By day 4, a*ngptl7* transcript abundance in intact skin was similar to time 0. Comparison of a*ngptl7* transcripts in intact and regenerating skin of the same individual at each time point analysed revealed significant down-regulation (*p* < 0.001) of *angptl7* in regenerating skin 1 and 3 days after scale removal.

Expression of *vegfab* in intact and regenerating skin was not significantly different (*p* > 0.05) at any time point analysed. In intact skin the expression of *vegfab* transcripts increased progressively and was significantly up-regulated (*p* = 0.0015) from day 3 onwards relative to skin at time 0 (Fig. 10f). Pairwise comparisons of *vegfab* transcripts in intact and regenerating skin of the same individual at each time point analysed did not reveal any significant differences. No correlation between *vegfab* and *angptl* expression was found (Additional file 7: Table S3b).

## Discussion

**Fig. 11:**
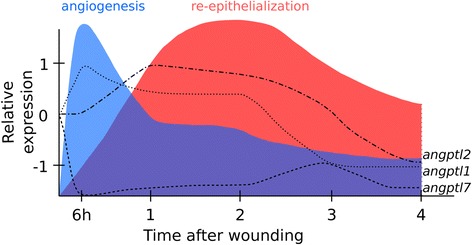
Summary of *angptl* gene expression during skin regeneration in sea bream. The x-axis indicates time after wounding (hours and days) and the y-axis relative expression of *angptl1b*, *angptl2b* and *angptl7*. Colours represent the approximate timing of angiogenesis and the re-epithelialization processes that overlap during the initial 4 days of wound healing in sea bream skin illustrated according to our morphometric evaluations (Figs. 8 and 9), in arbitrary units

The ANGPTL family is a large group of multifunctional proteins involved in skin regeneration and angiogenesis in mammals. The present study characterised for the first time the fish *Angptl* gene repertoire and identified the *Angptl* family members that were expressed in the integument of teleost fish and their expression during skin regeneration in the teleost sea bream. The results reveal that the involvement of ANGPTL family members in skin repair has been conserved during vertebrate evolution. In fish a new Angptl family member was found (*Angptl9*) and the orthologue of the mammalian *ANGPTL8* gene was lost. In teleost *angptl* genes duplicated as a consequence of the lineage specific genome doubling and some of the paralogues that persisted have a role in skin homeostasis. Orthologues of human *ANGPTL1* (*angptl1b*), *ANGPTL2* (*angptl2b*), *ANGPTL4* (*angptl4a*, *angptl4b*) and *ANGPTL7* that play a key role in mammalian angiogenesis, pro-inflammatory response, tissue re-epithelisation/cell proliferation and avascularity, respectively are expressed in sea bream skin. Variation in the abundance of *angptl1b*, *angptl2b* and *angptl7* during sea bream skin regeneration indicates that their role in tissue repair has been conserved (Fig. 11). The role in sea bream skin of the teleost *angptl4* paralogues that in mammals has a prominent role in skin repair, remains to be established.

### Angptl members in fish

Homologues of the mammalian ANGPTLs exist in fish. In the teleosts they have duplicated and phylogenetic analysis and gene synteny confirmed that this was the result of the lineage specific genome duplication [64]. Some of the gene duplicates have persisted and the ESTs retrieved for the *angptl* paralogues and their divergent *in silico* distribution in skin suggests that after genome duplication functional specialization occurred and that they acquired a range of different physiological functions. *Angptl* genes were found from lamprey (a jawless fish that diverged early from the vertebrate ancestral genome) to coelacanth (a lobe-finned fish that diverged subsequent to the teleosts and is basal to the tetrapods) to mammals. The Angptl family members from fishes shared highly conserved sequence and structural motifs with the human homologues and some of the residues of functional importance in the human protein have also been maintained [66]. The deduced proteins in fish and human share two highly conserved and characteristic signature motifs of this family: the N-terminal coiled-coil domain (CCD), which likely contributes to protein oligomerization, and the C-terminal fibrinogen-like domain (FReD) that in the related ANGPT protein family is a receptor-binding domain [68, 69]. A conserved N-terminus signal peptide in fish Angptls suggests that they are secreted and the identification of conserved potential N-glycosylation consensus sites in the predicted proteins suggests that like the mammalian orthologues they are glycosylated proteins and may function as pleotropic endocrine/autocrine factors [70]. In common with the human ANGPTLs, the fish members possess four conserved cysteine residues within the C-terminus FReD motif that potentially form two intramolecular disulphide bonds. However, the importance of the four highly conserved cysteine residues in the protein structure remains to be discovered as alanine replacement studies with human ANGPTL4 failed to reveal functional modifications [65]. ANGPT proteins possess a further two conserved cysteine residues compared to ANGPTL and the resulting conformational difference between the two sets of proteins is proposed to be the basis of receptor selectivity for the two protein families [70].

A number of changes have occurred during the evolution of the ANGPTL family and a new vertebrate member, Angptl9 was identified in the present study in non-mammalian vertebrates such as fish, the *Xenopus*, Anole lizard and chicken. Gene synteny analysis suggests that the loss of *ANGPTL9* from mammals was probably the result of chromosome rearrangements that occurred early in their radiation. Similarly, orthologues of the mammalian *ANGPTL8* gene were absent from the sequenced genomes of fish and other non-mammalian vertebrates. ANGPTL8 is an atypical family member as it lacks the FReD domain, the glycosylation sites and the amino acids forming the intramolecular disulphide bonds but it has overlapping functions with ANGPTL3 and 4 and inhibits lipoprotein lipase activity [24, 71]. The absence of an *ANGPTL8* gene in non-mammalian vertebrate genomes but the existence of a conserved gene environment across the vertebrates suggests that gene loss may have been due to lineage-specific deletions.

In mammals, the FReD is essential for the angiogenic activity of the ANGPTL protein while the CCD domain seems to be more important for other physiological functions, including regulation of lipid metabolism by inhibition of lipoprotein lipase [72]. The functional importance of Angptl proteins and their duplicates in fish physiology is unknown but given their high sequence homology with mammalian orthologues we hypothesized that their function might be conserved. In fact, in the zebrafish, the only teleost where members of this family have previously been described, the orthologues of human *ANGPTL1*, *2* and *6* are ubiquitously expressed and have a similar tissue distribution to human. The zebrafish *Angptl1* and *2* share a conserved role in vascular development with the human orthologues [42, 73].

Recently, Angptls were found to activate immune inhibitory receptors of the leukocyte immunoglobulin (Ig)-like family, a group of innate immune receptors, that are expressed on immune cells and involved in the control of inflammatory responses and cytotoxicity [30]. Studies of human ANGPTL2 and its receptor (LILRB2) indicate that neither the CCD nor the FReD binds to LILRB2. The authors suggest that ligand-receptor interactions occur via the receptors immunoglobulin domain and that ANGPTL2 protein multimerization is essential for downstream signalling [74]. Receptors for the fish Angptl are currently unknown, although potential immunoglobulin-like receptor transcripts have been described in fish and it will be important to establish if they have a similar role to those found in mammals [75].

### Angptl emerged early and evolved via gene duplications and deletions in the vertebrate radiation

In vertebrates, Angptl evolved via gene duplication and deletion events and they are proposed to have shared a common evolutionary origin with Angpt, with which they share sequence and structure similarities. Four main Angptl vertebrate protein clusters (Angptl1-2-6, Angptl3-4, Angptl5 and Angptl7-9) emerged from duplications of the ancestral *angptl* prior to the vertebrate radiation and the family members expanded during the early vertebrate genome doublings and segmental genome duplications [48, 76–79]. The teleosts are by far the most successful and diverse group of vertebrates with at least 28,000 species identified [17]. This success has been linked to the teleost specific genome duplication that is suggested to have provided the raw material for the evolutionary adaptations and innovation that are a characteristic of this group [80]. The phylogenetic analysis indicates that the *angptl* genes also duplicated early in the teleost radiation and this was followed by gene loss so that in extant teleost genomes only a few paralogues persisted [81]. In the coelacanth, species-specific gene duplication affected *Angptl5* and the duplicates map in tandem to the same genome fragment (data not shown) and clustering in the phylogenetic tree suggests that their sequence has diverged considerably from that in other vertebrates. The identification of only two *Angptl*s (*Angptl2* and *5*) in the lamprey and the non-identification of *Angptl5*, *6* and *9* in the cartilaginous fish genomes may be a consequence of their incomplete genome assemblies or linked to their unique physiological adaptations to an aquatic environment and associated gene deletions after their divergence from the common vertebrate ancestor.

Members of the Angptl family are suggested to have emerged prior to the vertebrate radiation and a sea squirt (*Ciona intestinalis*) gene homologue is equally similar to all the vertebrate members [82]. We have also found other putative *Angptl-like* genes in the genomes of several other early deuterostome species but their similarity with the vertebrate ANGPTL family was very low. The exception was in the cephalochordate (*Branchiostoma floridae*) genome where 5 *Angptl-like* genes were found. The cephalochordate Angptl-like deduced protein cluster revealed that four of the genes were orthologues of the vertebrate Angptl7. Furthermore, the conserved gene environment flanking the cephalochordate *Angptl-like* genes and the vertebrate *Angptl7* suggests that the ancestral *Angptl7* gene emerged prior to the vertebrate radiation. A putative *Angptl1-2-6-like* gene also seems to exist in the amphioxus and others genes of this family may potentially exist and their presence in the gene repertoire of the cephalochordate or in other early deuterostomes suggests this is an ancient gene family and their expansion in vertebrates may be a functional innovation linked to increased complexity of organisms and their physiology.

### Angptls in sea bream skin wound healing

In mammals’ skin wound healing is a complex and highly coordinated sequence of events. This process is triggered by blood clotting and is followed by inflammation, vascularization, formation of granular tissue and tissue remodelling [83]. In fish the sequence of events is different, re-epithelialization of damaged skin initiates immediately after wounding and the few studies of skin healing in fish reveals that this is independent of the inflammatory signals released by the blood clot [10, 84]. In all vertebrates during healing new blood vessels are formed from pre-existing ones in the dermis [83, 84] and in mammals’ new blood vessels are observed 3 days after wounding [83]. In teleosts this process is initiated much earlier and in zebrafish skin new blood vessels are observed 1 day after damage to the epidermis and dermis [84]. Similarly, in our sea bream skin regeneration model new blood vessels were observed 6 h after scale removal and did not correlate with a peak of *vegfab* (Additional file 7: Table S3c), an established vertebrate blood vessel maker, which reached maximal expression 1 day after wounding. In teleosts duplicate *vegfa* genes have been described, *vegfaa* and *vegfab*, and both can bind the Kdra and Kdrb (type III receptor tyrosine kinase) *in vitro*, participating in vascular development [67]. The involvement of the *vegfaa* paralogue in our sea bream skin healing model remains to be established and in the future characterisation of this and of other angiogenic factors (eg: angiopoietins) in tissue recovery will be of interest.

The expression in mammalian and teleost skins of *angptls*, their link with integument repair and specifically their co-ordinated appearance with key steps of the skin repair program in the sea bream suggests that they may share a conserved role in vertebrate skin regeneration. In sea bream *angptl1b*, *angptl2b*, *angptl4a*, *angptl4b* and *angptl7* are expressed in skin and their variable pattern of expression during wound healing and the differences in regenerating and intact skins 48 h post-wounding suggests they have acquired distinct roles in tissue re-epithelialization and angiogenesis. In zebrafish and mice *angptl1* and *angptl2* are suggested to stimulate angiogenesis and to promote endothelial cell apoptosis for the initiation of vascular development [41, 73]. In mice, *Angptl2* was also found to regulate the pro-inflammatory response and it activates resident murine peritoneal monocytes and macrophages [85]. We did not study markers of inflammation, however the increased expression of *angptl1b* and *angptl2b* was correlated with the number and diameter of blood vessel, respectively, during the early stages of skin regeneration in sea bream and suggests they also have a role in angiogenesis (Fig. 11 and Additional file 7: Table S3c).

In human *Angptl7* is an anti-angiogenic factor and *in vitro* studies using cornea keratocyte cells demonstrated that this gene may be responsible for maintaining tissue avascularity in the eye [36, 37]. The decreased expression of *angptl7* during sea bream skin regeneration and its negative correlation with blood vessel number suggests it may have a similar role to that in mammals (Fig. 11 and Additional file 7: Table S3c). In mammals, ANGPTL4 regulates skin re-epithelialization, its expression is increased during this process in mice [31] and in *Angptl4*-knockout mice there was decreased expression of genes involved in epidermal differentiation and cell proliferation in skin [33]. In murine ischemic tissues, ANGPTL4 bound to the ECM inhibits endothelial cell motility, sprouting and formation of new blood vessels [86]. The down-regulation of the duplicate *angptl4* genes during sea bream skin regeneration and their negative correlation with the thickness of epidermis and basement membrane (Additional file 7: Table S3c) suggests they are unlikely to be involved in re-epithelialization. Nonetheless, the time-frame of *angptl1b* and *angptl2b* up-regulation suggests they may be involved in this process (Fig. 11). The responsiveness of *angptl* family members in the intact skin of fish with a damaged flank was notable. In human and mammalian experimental models a systemic effect of local skin injuries, such as burns or diabetic wounds is well described in relation to cardiovascular and metabolic parameters [87, 88]. The results obtained for expression of *angptl1b*, *2b* and *7* in the intact skin of fish with a damaged flank suggests that it is the combination of both local and systemic effects that contribute to skin recovery; it will be important in the future to establish which cells are actively producing these proteins in skin and their role in teleost skin repair and homeostasis.

## Conclusions

The present study characterizes the homologues of the human ANGPTLs in fish and identifies the candidate members that are involved in teleost skin regeneration. In fish, amphibian and chicken a novel member of this family (*Angptl9*) was found but this gene is absent from mammals. Homologues of human *ANGPTL8* are not found in fish and they are only present in mammalian genomes. In the teleosts the *Angptl* family expanded and some gene paralogues acquired a role in the skin. In the sea bream, *angptl1b*, *angptl2b*, *angptl4a*, *angptl4b* and *angptl7* transcripts are present in skin but only *angptl1b*, *angptl2b* and *angptl7* are modified in response to damage and are presumably involved in skin repair. The change in abundance of *angptl1b* and *angptl2b* transcripts correlates with the timing of re-epithelialization and angiogenesis detected by histology. In contrast, *angptl7* is down-regulated and negatively correlated with angiogenesis suggesting that in common with mammals it is an anti-angiogenic factor (Fig. 11). Overall, the results indicate that Angptl family members are involved in sea bream skin homeostasis and repair.

## Additional files

Additional file 1: Table S1. Accession numbers of the fish, tetrapod and cephalochordate *Angptl* genes and sea bream transcripts. * very incomplete sequence and not included in the phylogenetic tree; ni-not identified. (XLSX 44 kb)
Additional file 2: Figure S1. Expanded phylogenetic tree of the fish and other metazoan ANGPTL family members generated using Bayesian Interference (BI). Details are available from Fig. 2. Accession numbers of the sequences used are given in the Additional file 1: Table S1 and Additional file 9: Table S5. (PPTX 197 kb)
Additional file 3: Figure S2. Phylogenetic tree of the fish and other metazoan ANGPTL family members constructed with the Maximum-likelihood (ML) algorithm. Analysis was performed in ATGC (http://www.atgc-montpellier.fr/phyml/) using the deduced amino acid sequence and a fixed value for the proportion of invariable sites 0.008, 4 gamma-distributed rate categories (1.272) and 100 bootstrap replicates according to ProtTest. Tree was rooted using the metazoan ANGPT clade (ANGPT1, 2 and 4). Accession numbers are given in the Additional file 1: Table S1 and Additional file 9: Table S5. (PPTX 215 kb)
Additional file 4: Figure S3. Sequence alignments of the human, spotted gar, zebrafish, sea bass and sea bream Angptls. Sequences were compared according to the clustering of the phylogenetic tree (Fig. 2, Additional file 2: Figure S1 and Additional file 3: Figure S2). a ANGPTL1-2-6; b ANGPTL3-4; c ANGPTL5 and d ANGPTL7-9. Conserved amino acids in the sequence alignment are shaded; dark grey represents 80% conservation and black 100% conservation. In the human sequences the signal peptide is underlined and in bold and the coiled-coil domain (CCD) are in bold and coloured blue. The conserved fibrinogen-related domain (FReD) in human and in fish is boxed in red and the four conserved cysteine residues within this motif that are involved in two intramolecular disulphide bonds are highlighted in yellow and the predicted glycosylation (N-x-T/S, where x represents any amino acid) motifs are highlighted in red. (PPTX 840 kb)
Additional file 5: Table S2. Percent of amino acid sequence identity/similarity of the fish Angptl family members with the human orthologues. (XLSX 39 kb)
Additional file 6: Figure S4. Sequence alignment of the deduced cephalochordate Angptl-like 7 protein with the human and spotted gar ANGPTL7. Conserved amino acids in the sequence alignment are shaded; dark grey represents 80% conservation and black 100% conservation. The coiled-coil domain (CCD) is coloured in blue for the human sequence. The conserved fibrinogen-related domain (FReD) is boxed and the four conserved cysteine residues within this motif that are potentially involved in the establishment of two intramolecular disulphide bonds of the vertebrate proteins are highlighted in yellow. (PPTX 149 kb)
Additional file 7: Table S3. Correlation analysis of gene expression profile and changes in skin morphology during the initial phases of piscine skin regeneration (thickness of the epidermis, basement membrane and dermis, number and diameter of the blood vessels). a, Skin parameters during sea bream skin regeneration (diameter and number of blood vessels, thickness of the epidermis, basement membrane and dermis); b, gene expression and c, gene expression and skin parameters. Represented in the tables are the correlation value (r) and the *p*-value (r / *p*-value). Correlations are highlighted in red. (PPTX 42 kb)
Additional file 8: Table S4. List of the teleost *angptl* ESTs and their origin. Data was retrieved from the NCBI database using sea bass *angptl* members as the query. ESTs retrieved were those present in skin or from teleost fins and bony structures covered with skin. (XLSX 31 kb)
Additional file 9: Table S5. Accession numbers of the fish, tetrapod and cephalochordate *Angpt* genes and transcripts. ni-not identified. (XLSX 34 kb)

## Abbreviations

aa: Amino acid
AMP: Antimicrobial peptide
ANGPT: Angiopoietin
ANGPTL: Angiopoietin-like
CCD: Coiled-coil domain
ECM: Extracellular matrix
FGFR1: Fibroblast Growth Factor Receptor 1
FReD: Fibrinogen-related domain
Ig: Immunoglobulin
Kdr: Type III receptor tyrosine kinase receptor
LPL: Lipoprotein lipase
qPCR: Quantitative real-time polymerase chain reaction
TGFß: Transforming Growth Factor beta
vegfaa: Vascular endothelial growth factor aa
vegfab: Vascular endothelial growth factor ab
