# Supplementary figures and images for: Evolution of the angiopoietin-like gene family in teleosts and their role in skin regeneration
Source: BMC Evol Biol. 2017 Jan 13;17:14. doi: 10.1186/s12862-016-0859-x (PMC5237311; doi:10.1186/s12862-016-0859-x)

## Slide 1
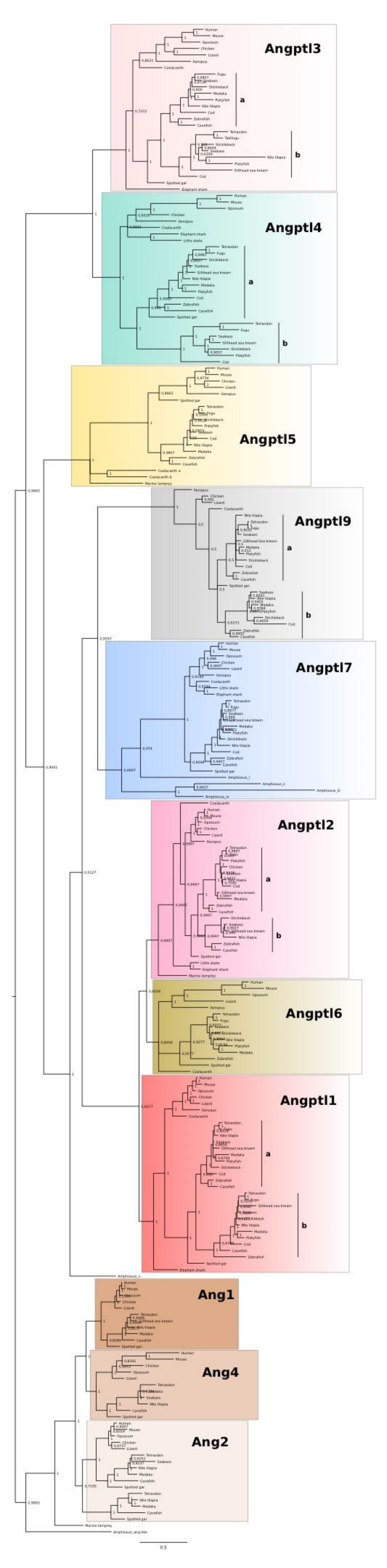

Supplement: Additional file 2: Figure S1. — Expanded phylogenetic tree of the fish and other metazoan ANGPTL family members generated using Bayesian Interference (BI). Details are available from Fig. 2. Accession numbers of the sequences used are given in the Additional file 1: Table S1 and Additional file 9: Table S5. (PPTX 197 kb) [file 12862_2016_859_MOESM2_ESM.pptx]

## Slide 1
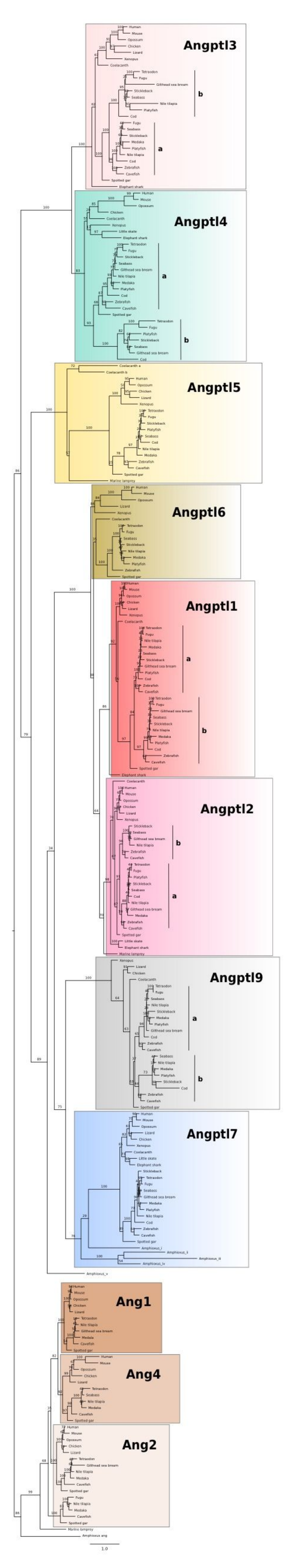

Supplement: Additional file 3: Figure S2. — Phylogenetic tree of the fish and other metazoan ANGPTL family members constructed with the Maximum-likelihood (ML) algorithm. Analysis was performed in ATGC (http://www.atgc-montpellier.fr/phyml/) using the deduced amino acid sequence and a fixed value for the proportion of invariable sites 0.008, 4 gamma-distributed rate categories (1.272) and 100 bootstrap replicates according to ProtTest. Tree was rooted using the metazoan ANGPT clade (ANGPT1, 2 and 4). Accession numbers are given in the Additional file 1: Table S1 and Additional file 9: Table S5. (PPTX 215 kb) [file 12862_2016_859_MOESM3_ESM.pptx]

## Slide 1
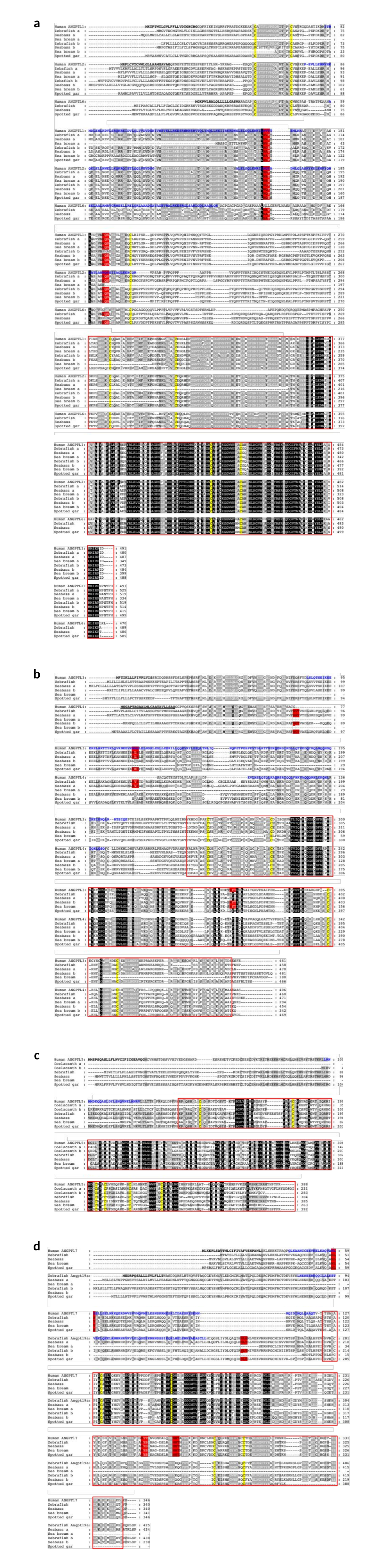

a
b
c
d

Supplement: Additional file 4: Figure S3. — Sequence alignments of the human, spotted gar, zebrafish, sea bass and sea bream Angptls. Sequences were compared according to the clustering of the phylogenetic tree (Fig. 2, Additional file 2: Figure S1 and Additional file 3: Figure S2). a ANGPTL1-2-6; b ANGPTL3-4; c ANGPTL5 and d ANGPTL7-9. Conserved amino acids in the sequence alignment are shaded; dark grey represents 80% conservation and black 100% conservation. In the human sequences the signal peptide is underlined and in bold and the coiled-coil domain (CCD) are in bold and coloured blue. The conserved fibrinogen-related domain (FReD) in human and in fish is boxed in red and the four conserved cysteine residues within this motif that are involved in two intramolecular disulphide bonds are highlighted in yellow and the predicted glycosylation (N-x-T/S, where x represents any amino acid) motifs are highlighted in red. (PPTX 840 kb) [file 12862_2016_859_MOESM4_ESM.pptx]

## Slide 1
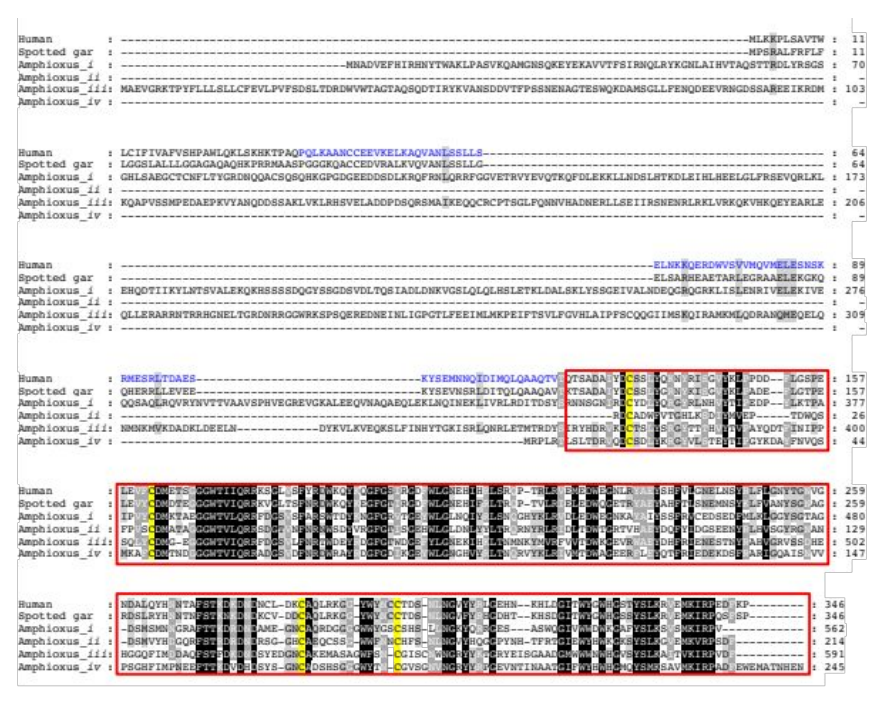

Supplement: Additional file 6: Figure S4. — Sequence alignment of the deduced cephalochordate Angptl-like 7 protein with the human and spotted gar ANGPTL7. Conserved amino acids in the sequence alignment are shaded; dark grey represents 80% conservation and black 100% conservation. The coiled-coil domain (CCD) is coloured in blue for the human sequence. The conserved fibrinogen-related domain (FReD) is boxed and the four conserved cysteine residues within this motif that are potentially involved in the establishment of two intramolecular disulphide bonds of the vertebrate proteins are highlighted in yellow. (PPTX 149 kb) [file 12862_2016_859_MOESM6_ESM.pptx]
