# Supplementary material for: Evolution of the angiopoietin-like gene family in teleosts and their role in skin regeneration
Source: BMC Evol Biol. 2017 Jan 13;17:14. doi: 10.1186/s12862-016-0859-x (PMC5237311; doi:10.1186/s12862-016-0859-x)
Supplement: Additional file 7: Table S3. — Correlation analysis of gene expression profile and changes in skin morphology during the initial phases of piscine skin regeneration (thickness of the epidermis, basement membrane and dermis, number and diameter of the blood vessels). a, Skin parameters during sea bream skin regeneration (diameter and number of blood vessels, thickness of the epidermis, basement membrane and dermis); b, gene expression and c, gene expression and skin parameters. Represented in the tables are the correlation value (r) and the p-value (r / p-value). Correlations are highlighted in red. (PPTX 42 kb) [file 12862_2016_859_MOESM7_ESM.pptx]

## Slide 1
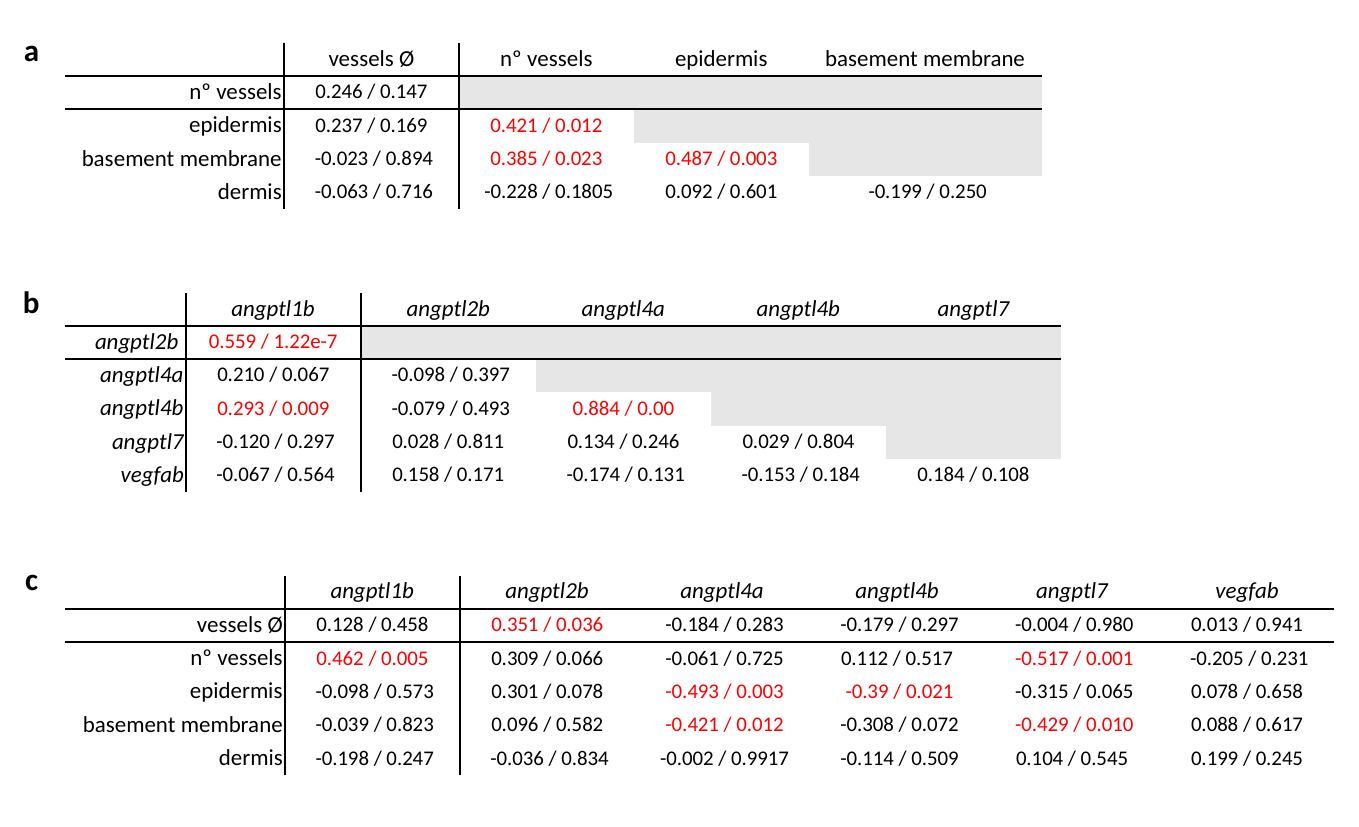

a
| | vessels Ø | nº vessels | epidermis | basement membrane |
| --- | --- | --- | --- | --- |
| nº vessels | 0.246 / 0.147 | | | |
| epidermis | 0.237 / 0.169 | 0.421 / 0.012 | | |
| basement membrane | -0.023 / 0.894 | 0.385 / 0.023 | 0.487 / 0.003 | |
| dermis | -0.063 / 0.716 | -0.228 / 0.1805 | 0.092 / 0.601 | -0.199 / 0.250 |
b
| | angptl1b | angptl2b | angptl4a | angptl4b | angptl7 |
| --- | --- | --- | --- | --- | --- |
| angptl2b | 0.559 / 1.22e-7 | | | | |
| angptl4a | 0.210 / 0.067 | -0.098 / 0.397 | | | |
| angptl4b | 0.293 / 0.009 | -0.079 / 0.493 | 0.884 / 0.00 | | |
| angptl7 | -0.120 / 0.297 | 0.028 / 0.811 | 0.134 / 0.246 | 0.029 / 0.804 | |
| vegfab | -0.067 / 0.564 | 0.158 / 0.171 | -0.174 / 0.131 | -0.153 / 0.184 | 0.184 / 0.108 |
c
| | angptl1b | angptl2b | angptl4a | angptl4b | angptl7 | vegfab |
| --- | --- | --- | --- | --- | --- | --- |
| vessels Ø | 0.128 / 0.458 | 0.351 / 0.036 | -0.184 / 0.283 | -0.179 / 0.297 | -0.004 / 0.980 | 0.013 / 0.941 |
| nº vessels | 0.462 / 0.005 | 0.309 / 0.066 | -0.061 / 0.725 | 0.112 / 0.517 | -0.517 / 0.001 | -0.205 / 0.231 |
| epidermis | -0.098 / 0.573 | 0.301 / 0.078 | -0.493 / 0.003 | -0.39 / 0.021 | -0.315 / 0.065 | 0.078 / 0.658 |
| basement membrane | -0.039 / 0.823 | 0.096 / 0.582 | -0.421 / 0.012 | -0.308 / 0.072 | -0.429 / 0.010 | 0.088 / 0.617 |
| dermis | -0.198 / 0.247 | -0.036 / 0.834 | -0.002 / 0.9917 | -0.114 / 0.509 | 0.104 / 0.545 | 0.199 / 0.245 |
